# Carrier Dynamics and Recombination Pathways in Ag–In–Zn–S Quantum Dots

**DOI:** 10.1021/acs.jpclett.4c02126

**Published:** 2024-10-11

**Authors:** Adam Ćwilich, Daria Larowska-Zarych, Patrycja Kowalik, Kamil Polok, Piotr Bujak, Magdalena Duda, Tomasz Kazimierczuk, Wojciech Gadomski, Adam Pron, Łukasz Kłopotowski

**Affiliations:** † Institute of Physics, Polish Academy of Sciences, 02-668 Warsaw, Poland; ‡ Faculty of Chemistry, University of Warsaw, 02-089 Warsaw, Poland; ¶ Faculty of Chemistry, Warsaw University of Technology, 00-664 Warsaw, Poland; § Faculty of Physics, University of Warsaw, 02-093 Warsaw, Poland

## Abstract

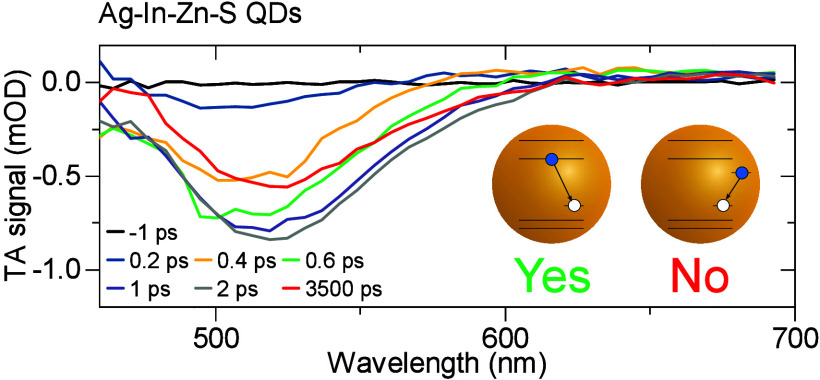

Strong tolerance to off-stoichiometry of group I–III–VI semiconductors in their nanocrystal form allows fabrication of multinary, alloyed structures of desired properties. In particular, alloyed Cu–In–Zn–S and Ag–In–Zn–S quantum dots (QDs) have recently emerged as efficient fluorophors, in which tailoring the composition allows tuning the optical properties, and achieving photoluminescence (PL) quantum yields approaching unity. However, poor understanding of the carrier recombination mechanism in these materials limits their further development. In this work, by studying transient absorption and temperature dependent PL on bare QDs and QDs conjugated with electron scavenger molecules, we obtain a detailed picture of carrier dynamics. Our results challenge the prevailing assumption that the PL is due to a donor–acceptor-pair transition. We show that the PL occurs as a result of a recombination of a delocalized electron with a localized hole.

Colloidal semiconductor quantum dots (QDs) are exciting fluorophors, envisioned as building blocks of many optoelectronic devices and already applied in commercially available displays.^1^ The most thoroughly studied QD materials are cadmium and lead chalcogenides and lead halide perovskites. However, concerns related to the inherent toxicity of Pb and Cd ions inspire a quest for alternatives.^2,3^ Recent years have seen a rapid development of QDs based on I–III–VI compounds, such as CuInS_2_ or AgInS_2_.^4−7^ The critical advantage of these QDs over the commonly studied lead and cadmium compounds is a wider range of QD architectures, which can be achieved and used to tailor the optical properties. Just like the binary compounds, these QDs can be synthesized in the form of core-only and core–shell structures (such as CuInS_2_/CdS or AgInS_2_/ZnS). However, importantly, the I–III–VI-based QDs exhibit a much larger tolerance for off-stoichiometry than the binary compounds. This property together with the possibility of alloying with, e.g., ZnS opens a possibility to achieve quaternary compounds such as Ag–In–Zn–S or Cu–In–Zn–S with a huge range of possible compositions and morphologies.^6,8^ Tailoring the alloyed QD composition allowed PL quantum yields to be achieved routinely above 50%^9,10^ and tuning the absorption and PL spectra.^11^

Photoluminescence properties of the quaternary QDs are analogous to those reported for ternary CuInS_2_ and AgInS_2_ QDs. Namely, the Stokes shifts between the PL peaks and absorption onsets are on the order of 500 meV resulting in negligible reabsorption losses—a property ideal for applications as luminescent solar concentrators.^12,13^ The PL line widths are on the order of several hundred meV, which can be exploited in solid state lighting.^7^ PL lifetimes are as long as hundreds of nanoseconds to microseconds, which—together with the tunability of absorption spectra—make the quaternary QDs promising for fluorescence lifetime imaging and photocatalysis.^14−16^ Moreover, the PL lifetimes exhibit a spectral dependence: the PL decay rate increases with the PL energy.^17−20^

All of the above-mentioned properties are analogous to those of bulk semiconductors exhibiting a donor–acceptor-pair (DAP) transition–recombination of an electron and a hole localized at a donor and an acceptor defect, respectively, see Figure 1(a).^21^ Based on this circumstantial evidence, it is widely assumed that in quaternary QDs the DAP mechanism is responsible for the PL.^11,17−19,22−24^ However, even the proponents of this conclusion recognize that the DAP model does not fully explain the PL features, e.g., the strong dependence of the PL energy on the QD size.^6^ Therefore, the question of the PL mechanism in quaternary QDs is far from resolved. Notably, early studies of CuInS_2_ and AgInS_2_ QDs also assumed DAP recombination as the PL mechanism^17,25^ until more advanced spectroscopic studies revealed that the luminescent excited state consists of an electron delocalized on the quantized state in the conduction band and a localized hole. Thus, the PL occurs via a free-to-bound mechanism, see Figure 1(b).^26−30^ This knowledge allowed to tailor the deexcitation pathways in CuInS_2_ QDs offering a detailed control of the emission process.^31,32^ Moreover, as proven crucial in the development of CdSe QDs, detailed understanding of the PL mechanism leads to QD designs that allow the rates of radiative and nonradiative recombination to be tuned,^33^ blinking to be suppressed,^34^ the emissive state structure to be controlled,^35^ and charge carrier doping to be achieved.^36^ Thus, the assumption that DAP recombination is responsible for PL in quaternary QDs limits attempts at further development and tailoring of the PL properties. The electron and hole trapping at donor and acceptor defects, which presumably occurs in quarternary QDs, is an extrinsic process, i.e., difficult to control. The tolerance to off-stoichiometry in ternary and quarternary QDs goes in hand with a proneness to formation of point defects—vacancies, antisites, and interstitials.^5,17,24,28^ As a result, the prevailing view in the literature is that the PL occurring as a result of DAP recombination is a natural consequence of the rich defect chemistry of I–III–VI and related QDs.

**Figure 1:**
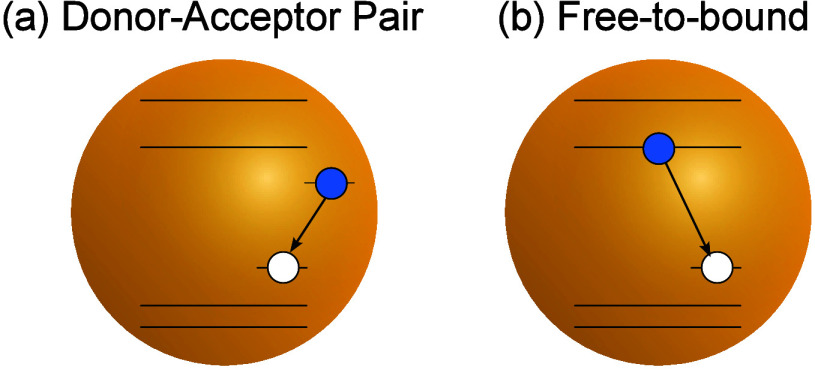
Schemes of the recombination pathways discussed in the text. Long (short) horizontal lines denote delocalized quantum states (localized optically active trap states). (a) Donor–acceptor-pair mechanism: recombination of a localized electron with a localized hole. (b) Free-to-bound mechanism: recombination of a delocalized electron with a localized hole.

In this work, we study room temperature transient absorption (TA) and temperature dependent PL dynamics on bare QDs and QDs conjugated with electron scavenger molecules. The results allow us to achieve a detailed understanding of the carrier dynamics of these materials. In TA studies on bare QDs, we observe an exciton bleach indicating the presence of long-lived delocalized electrons and a distinct photoinduced absorption signal which we attribute to holes localized at midgap states. The PL measurements show that lowering the temperature results in a decrease in the PL decay rate, which we associate with a dark-bright splitting of the luminescent excited state. We argue that these observations are incompatible with the DAP mechanism of the PL. Instead, these results indicate that in the studied samples, the delocalized electrons recombine with localized holes. We show that this conclusion agrees with theoretical considerations and discuss conditions that have to be fulfilled for the DAP mechanism to be possible.

Our studies are performed on two samples. Sample A1 consists of spherical Ag–In–Zn–S QDs with an average diameter of ∼3.7 nm. Sample A2 contains rod-shaped QDs with a similar average diameter of ∼3.9 nm and a length of ∼9.0 nm. The details of the synthesis procedure are given in the Supporting Information, Section S1. The results of structural studies are shown in the Supporting Information, Section S2, and reported in detail in ref (11). Briefly, as shown by X-ray diffraction studies and high-resolution transmission electron microscopy, the QD crystal structure forms an alloy of orthorhombic AgInS_2_ and wurtzite ZnS. No other phases were detected. The chemical compositions for A1 and A2, determined via energy-dispersive X-ray spectroscopy, can be written as AgIn_1.5_Zn_1.9_S_3.6(4.6)_ and AgIn_1.5_Zn_4.4_S_6.8(7.1)_, respectively. The numbers in parentheses denote the relative sulfur molar fractions assuring charge neutrality. Hence, we see that the QDs are cation-rich. Despite the strong nonstoichiometries, these QDs exhibit PL QY of 40% and 18% for sample A1 and A2, respectively.

Absorption and PL spectra of sample A1 are plotted in Figure 2(a). Typically for ternary and quaternary QDs, the absorption spectrum is essentially featureless with no clear edge. The PL spectrum is peaked at about 1.9 eV (650 nm), significantly red-shifted with respect to the absorption onset. The full width at half-maximum (fwhm) line width is 0.57 eV. PL decays measured at different detection energies are presented in Figure 2(b). Average PL lifetimes, evaluated by fitting a triple-exponential decay function, are plotted against the PL spectrum in Figure 2(a). A clear shortening of the PL lifetime with decreasing PL wavelength is observed: at the low energy tail of the PL spectrum the lifetime is as long as 3 μs, and it decreases to 1 μs at the PL peak and further decreases to 0.5 μs at the high energy tail. Analogous properties are exhibited by sample A2, as shown in the Supporting Information, Figure S3. The long PL lifetimes reflect the high PL QYs of both samples and overall the properties shown in Figures 2(a) and (b) are typical for alloyed quarternary QDs.^6,9−11,19,20,24^

**Figure 2:**
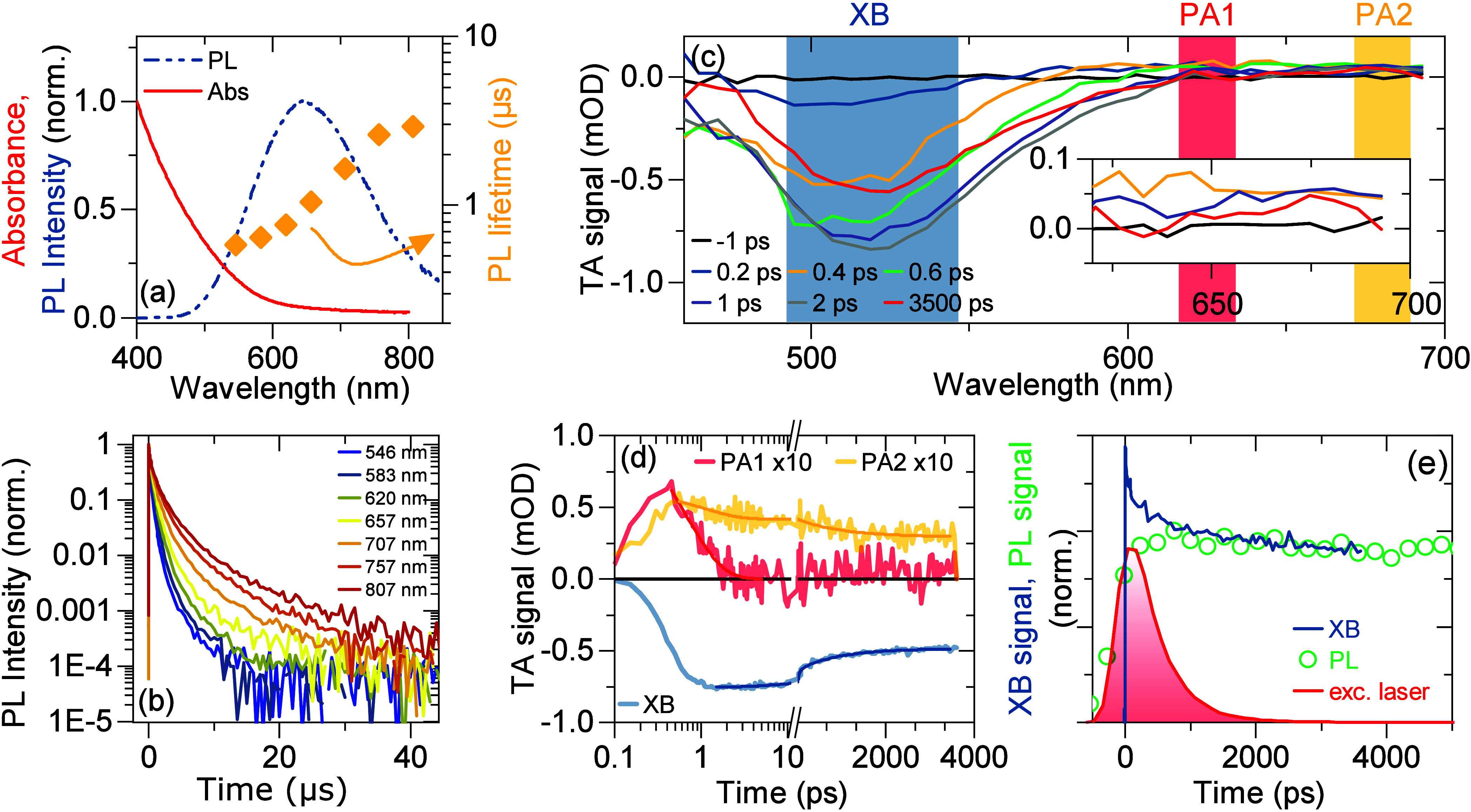
(a) Absorption (solid line) and PL (broken line) spectra of sample A1. Yellow diamonds denote detection wavelength dependence of average PL lifetimes. (b) Normalized PL decays of sample A1 measured at various detection wavelengths given in the legend. (c) Transient absorption spectra for sample A1 for various pump–probe delays indicated in the legend. The inset shows a magnification of the red edge of the spectra. (d) TA kinetics for three signals denoted in (c): ground state bleach (XB, blue) and two photoinduced absorption bands (PA1—red and PA2—yellow). Thick semitransparent lines denote experimental transients, and thin lines denote fits of eq 2. Note that PA1 and PA2 transients were multiplied by 10 to enable the comparison with the XB transient. (e) Comparison of TA kinetics (blue line) and PL dynamics (green open points). Red shaded area denotes the temporal profile of the laser used for PL excitation.

Importantly, all of the above properties have, to date, been attributed to the DAP recombination mechanism depicted schematically in Figure 1(a). The model assumes that after interband photoexcitation the electrons and holes are trapped at donor and acceptor defect sites, respectively. The energy of the emitted photons *E*_*DAP*_ is then given by^21^
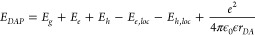
1where *E*_*g*_ is the bulk semiconductor band gap and *E*_*e*_ and *E*_*e*,*loc*_ (*E*_*h*_ and *E*_*h*,*loc*_) are the energies of electron (hole) quantized states and localization energies, respectively. The last term describes the Coulomb interaction between the ionized donor and acceptor defects present in the final state of the PL transition. In this term, ϵ_0_ and ϵ are, respectively, the vacuum and QD material permittivity, and *r*_*DA*_ is the donor–acceptor distance. In the DAP model, the large Stokes shifts are attributed to the electron and hole localization energies and the large PL line widths to their ensemble distributions. The absorption (“Urbach”) tails are assigned to a large concentration of defects, inducing absorption transitions to the respective band edge states. The crucial argument in favor of the DAP model is the spectral dependence of the PL lifetimes. Arguably, the decays at, respectively, high and low energies, probe donor–acceptor pairs closer and further apart, see eq 1, with larger and smaller electron–hole wave function overlaps and, hence, larger and smaller PL decay rates, respectively. In the following, we challenge the interpretation of the PL properties in terms of the DAP mechanism and demonstrate spectroscopic evidence that the PL transition occurs between an electron delocalized in a quantized state and a localized hole.

The TA experiments are carried out on samples dispersed in chloroform in 0.2 cm cuvettes. The QDs are excited with a ∼100 fs pump laser pulse at 400 nm with excitation densities ranging from 63 to 1840 μJ/cm^2^. A broadband white light supercontinuum probe pulse is used to monitor the changes in sample absorption in the 450–690 nm range, at variable time delays with respect to the pump. Details of the TA setup are given in the Supporting Information, Section S1. Let us start with the discussion of data acquired at the lowest excitation density corresponding to a fluence of 1.3 × 10^14^ photons per cm^2^. Under this condition, the average number of excitons per QD is about 0.18 (see the Supporting Information, Section S4). In Figure 2(c), we plot TA spectra collected for sample A1 at different time delays between the pump and probe pulses. Despite the featureless steady-state absorption, the TA spectra clearly reveal a ground state bleach (XB) centered at 520 nm (2.38 eV) with a fwhm line width of about 0.25 eV and a photoinduced absorption (PA) signal extending from ∼600 nm (2.07 eV) toward the near-infrared. Crucially, the XB at the short wavelength (high energy) side develops and decays quicker than the XB signal at the long wavelength (low energy) side, see Supporting Information, Figure S5. This behavior is consistent with carrier cooling—a stepwise energy relaxation down the ladder of electron and/or hole quantized states.^37^ Due to inhomogeneous broadening, the bleaching of transitions related to these states merges into one broad XB feature. The cooling is thus observed as its transient redshift. The picosecond dynamics of this shift reflects a superposition of size-dependent electron cooling. We note that XB cannot be associated with a saturation of the Ag^+^(4d^10^) → *e*_*CB*_ + Ag^2+^(4d^9^) transition: one lifting an electron from the filled silver 4d^10^ shell to the CB, observed in Ag-doped CdSe QD.^38^ For this transition, we expect neither a spectral dependence of the dynamics nor saturation of ns bleach signal at high excitation powers (see Supporting Information, Figure S4). Therefore, we assign the XB signal to electrons and/or holes occupying *delocalized* quantized states of the nanocrystals.^2^

Temporal decay of the XB signal, shown in Figure 2(d), allows us to monitor the dynamics of the delocalized carrier population. The transient can be well fitted with a double exponential decay function plus a constant:
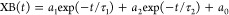
2The two exponentials describe a decay of the delocalized carrier population within the time window of our measurements (3.6 ns), while the *a*_0_ term indicates that a part of this population lives significantly longer. Since under our excitation conditions the probability of finding a QD occupied with more than 1 exciton is less than 0.015, we can rule out nonradiative Auger recombination as the mechanism of carrier loss. We therefore assume that the partial decay of XB monitors carrier trapping. The parameters obtained by fitting the XB decay with eq 2 are given in Table 1. The obtained values indicate that about η_*e*_ = (*a*_1_ + *a*_2_)/(*a*_1_ + *a*_2_ + *a*_0_) = 36% of delocalized carriers is trapped on a sub-ns time scale. More importantly, the above results indicate that a majority (∼64%) of delocalized carriers live much longer than our measurement window of 3.6 ns.

**Table 1:** Parameters Obtained from Fitting the XB and PA2 Transients with Equation 2

	*a*_0_ (10^–4^ OD)	*a*_1_ (10^–4^ OD)	*a*_2_ (10^–4^ OD)	τ_1_ (ps)	τ_2_ (ps)
XB	–4.84 ± 0.04	–1.21 ± 0.06	–1.54 ± 0.06	36 ± 5	907 ± 84
PA2	0.29 ± 0.02	0.22 ± 0.06	0.12 ± 0.02	1.02 ± 0.33	1030 ± 413

Before identifying whether electrons, holes, or both contribute to the XB signal, let us discuss the implications of the observations described above for the PL mechanism in the quaternary QDs. The key point is that the recombination probability for delocalized carriers is larger than that for localized ones. Therefore, if the PL were governed by the DAP mechanism, it should exhibit a rise dynamics occurring on ≫4 ns time scale, reflecting the decay of the delocalized carrier population via trapping at donor and acceptor defects. In Figure 2(e), we compare the XB dynamics with the initial PL dynamics. Crucially, the PL signal rises on a ≲ 100 ps time scale, i.e., a similar time-scale as the excitation laser. The presence of the long-lived delocalized carrier population and the lack of a slow rise dynamics in the PL transient rule out the DAP recombination as the PL mechanism. Instead, the observations presented above indicate that the PL process involves delocalized carriers.

In order to identify which carriers are responsible for the XB signal, we study TA of sample A1 with the QDs conjugated with methyl viologen (MV^2+^) molecules, a sample hereafter labeled as A1+MV (see the Materials and Methods section for the procedure). MV^2+^ is a well-known electron scavenger routinely used for extraction of electrons from photoexcited QDs.^39−41^ In Figure 3(a), we compare the steady state absorption spectra for the A1 and A1+MV samples. We find a negligible difference indicating that the absorption properties of the QDs remain intact after conjugation. On the other hand, adsorption of MV^2+^ on the QD surface results in complete disappearance of the PL signal. The TA spectra for sample A1+MV, shown in Figure 3(b), exhibit a much weaker XB signal compared to A1 (conf. Figure 2(c)). Transient redshift of the XB feature is clearly observed, but the bleach disappears before cooling is complete. Moreover, a PA band with a maximum at 625 nm is observed. The transients for these two signals are presented in Figure 3(c). Clearly, the XB signal disappears in ∼1 ps. We attribute the ultrafast bleach recovery to transfer of photoexcited electrons to the MV^2+^ molecules. Therefore, we associate the XB signal with filling of delocalized electron states with a negligible contribution from the hole states. Electron extraction outcompetes the cooling, and as a result, the electrons are removed before reaching the ground state. Consequently, the PL is completely quenched. Electron transfer leads to reduction of the methyl viologen molecules to MV^+^. The fingerprint of this process is the ultrafast appearance of the PA band at 625 nm.^39−41^

**Figure 3:**
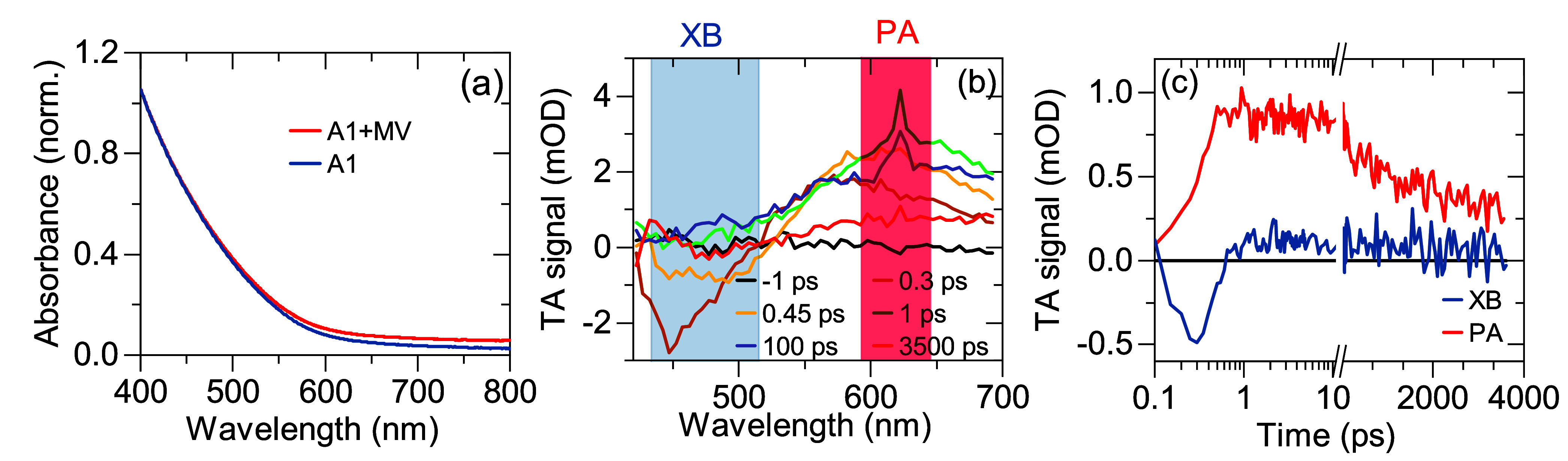
(a) Steady-state absorption spectra for sample A1 and A1-MV, where the QDs are conjugated with methyl viologen (MV^2+^) molecules. (b) Transient absorption spectra for sample A1-MV for various pump–probe delays indicated in the legend (the narrow peak on top of PA signal is an artifact). (c) TA kinetics for XB and PA signals denoted in (b).

The results presented above show that delocalized electrons participate in the PL process. To gain more insight into the nature of the luminescent excited state, let us now analyze what happens to the photoexcited holes. To this end, we analyze the dynamics of the PA signal recorded for sample A1 and presented in Figure 2(c) and (d).

The PA signal consists of two spectrally overlapping components: a short-lived absorption, PA1, best visible around 625 nm at pump–probe delays up to ∼1 ps, and a long-lived component, PA2, developing in less than 1 ps and extending from ∼630 nm toward the infrared (see Figures 2(c) and the Supporting Information Figure S3 and Section S6). Following other authors, we attribute PA1 to pump-induced biexciton absorption in the presence of hot electrons.^42−45^ The PA1 signal decays with a characteristic time of (0.66 ± 0.06) ps, which can be attributed to electron cooling.^46^ The PA2 signal, which persists for significantly longer than 3.6 ns, can originate from intraband transitions or from transitions involving carriers trapped on optically active midgap states.^47,48^ As we argued above, the hole contribution to the ground state bleach is negligible. Hence, the holes have to experience ultrafast trapping.^2^ Thus, an influence of intraband hole transitions on PA2 is ruled out. Likewise, on the basis of different behaviors of power dependence of XB and PA2 transients, intraband electron transitions are ruled out, see Supporting Information, Section S4. Furthermore, since the PA2 signal develops on the subpicosecond time scale, it cannot arise from electron trapping, which occurs on a subnanosecond time scale; see discussion above and Table 1. We therefore assign the PA2 signal to trapped holes. This interpretation is supported by analysis of the PA signal recorded for A1-MV sample. As seen in Figure 3(b), the band attributed to absorption by (reduced) MV^+^ molecules disappears at long pump–probe delays, and at 3.5 ns the PA structure in the TA spectrum resembles the PA2 signal observed for A1 sample. We assume that the holes localize at silver vacancy defects prevalent in alloyed Ag–In–Zn-S QDs^24^ or at orbitals neighboring silver in analogy to hole localization in Ag-doped CdSe QDs.^49^ Analogous PA signal attributed to trapped holes was observed for CuInS_2_ and Cu-doped CdSe QDs (with the hole localizing at the Cu d orbital),^50,51^ in Cu–In–Zn–S quaternary QDs,^52^ and in CdS nanorods.^53^

To monitor the population dynamics of trapped holes, we analyzed the PA transient at 680 nm, shown in Figure 2(d). At this wavelength there is still some overlap between the PA1 and PA2 transients. The signal can be well fitted with eq 2. Fit parameters are given in Table 1. The short decay time, *t*_1_ ≈ 1 ps, is within the fitting error equal to the PA1 decay time evaluated for the PA signal at 625 nm. We therefore attribute *t*_1_ to the PA1 decay. Similarly to electron dynamics monitored by the XB signal, we find that a part of the midgap hole population is lost on a sub-ns time scale. Since the *a*_1_ amplitude is ascribed to PA1, the fraction of lost holes is calculated as η_*h*_ = *a*_2_/(*a*_2_ + *a*_0_) = 0.30.

The analysis presented above provides a detailed picture of carrier dynamics in Ag–In–Zn–S QDs. Following photoexcitation with high excess energy, the electrons first cool down in ∼0.5 ps to a delocalized, quantized ground state in the CB. Afterward, the electron population decays via two competing mechanisms: radiative recombination and trapping on nonemissive states. Within 3.6 ns, a fraction equal to η_*e*_ = 0.36 of the electron population is trapped. Comparing the untrapped fraction 1 – η_*e*_ = 0.64 to the PL QY of 0.4 implies that electron trapping exhibits distributed kinetics beyond our time window of 3.6 ns. Photoexcited holes become localized at midgap absorptive states on a sub-ps time scale. These holes participate in radiative recombination with electrons. However, similarly to the delocalized electron population, a fraction η_*h*_ of the hole population is removed to the optically inactive traps. In short, we conclude that the PL process in alloyed Ag–In–Zn–S QDs is governed by a recombination of delocalized electrons with localized holes, while nonradiative recombination is due to trapping, most likely at surface states. Within this picture, the common PL properties of alloyed QDs, shown in Figure 2, can be explained. The large Stokes shifts are a consequence of hole localization at intragap states. Large PL line widths are attributed to a distribution of localization energies and a strong electron–phonon coupling. Finally, the spectrally dependent PL decays reflect a correlation between localization energies and positions: traps located closer to the surface are deeper due to an enhanced electron–phonon coupling.^54^ The electron–hole wave function overlap for localization closer to the surface is smaller than for holes localized at the QD center leading to longer and shorter lifetimes, respectively.

The conclusion regarding the recombination mechanism is supported by studies of PL dynamics. In Figure 4(a), we plot a spectrally integrated room temperature PL transient. Clearly, the PL dynamics is multiexponential in agreement with other reports^11,19,20^ and with spectrally resolved data shown in Figure 2(b). We fit the measured transient with a model developed by Hinterding et al. that takes into account a distribution of lifetimes over the QD ensemble.^29^ More specifically, we calculate radiative transition rates assuming that hole localization sites are uniformly distributed over a volume of sphere with a radius *r*_*max*_ < *a*, where *a* is the QD radius. This approach assumes negligible recombination probability for sites close to the surface.^55,56^ As a result, the PL dynamics is given by^29^
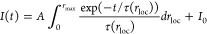
3where *A* is the signal amplitude, *I*_0_ is the background, and *r*_*loc*_ is the distance to the localization site from the QD center. The localization site dependent decay time is given by^29^
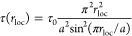
4We obtain an excellent agreement between the experimental and fitted transients with only four fit parameters: *A*, *I*_0_, *r*_*max*_, and minimum PL lifetime, τ_0_, corresponding to electron recombination with a hole at the QD center. In the Supporting Information, Figure S8, we show that eq 3 fits the data better than a biexponential decay (5 fit parameters) and triexponential decay (7 fit parameters). This observation indicates that the model curve (eq 3) accounts for more variance in the PL signal than the generic approaches.

**Figure 4:**
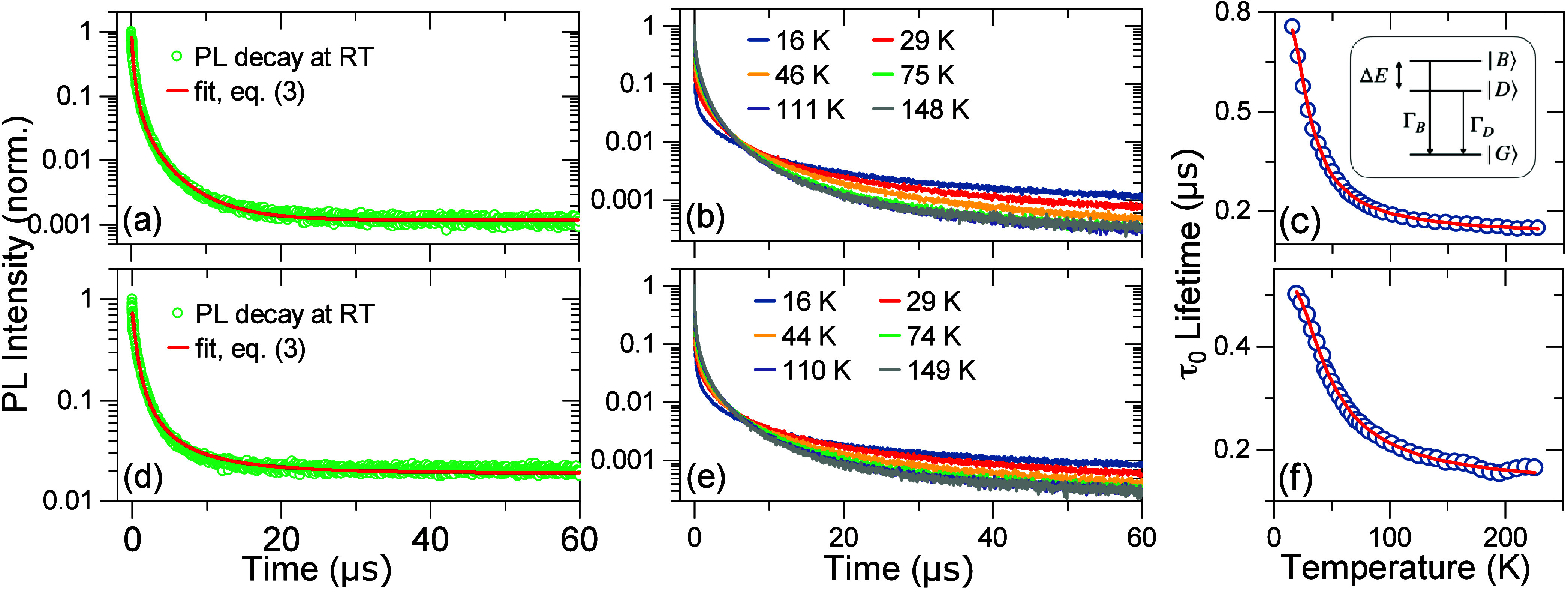
Temperature-dependent PL dynamics measured for samples A1 and A2. (a) Open points: PL transient measured at room temperature for sample A1. Line: fit of the model incorporating homogeneous distribution of hole localization sites, eq 3. See text for details. (b) PL transients measured at various temperatures indicated in the legend. (c) Open points: Temperature dependence of the PL lifetime parameter τ_0_ obtained by fitting the temperature dependent PL transients with eq 3. Line: Fit of a model assuming a luminescent excited state depicted in the inset in thermal equilibrium, eq 5. In the inset, |*G*⟩ denotes the QD ground state, while |*B*⟩ and |*D*⟩ denote bright and dark excited states split by energy Δ*E*. Γ_*B*_ and |Γ_*D*_ are radiative rates from the bright and dark states, respectively. (d), (e), and (f) present analogous data for sample A2.

To gain more insight into the nature of the luminescent state, we measured the PL dynamics as a function of temperature. The PL transients measured in the range between ∼15 and ∼150 K are plotted in Figure 4(b). In this temperature range, we observe an initial, short decay component visible up to ∼40 ns and a long decay component, which accelerates as the temperature increases. The amplitude of the fast component decreases with temperature indicating thermalization effects.^57,58^ We perform fitting of long decays with eq 3 starting at a delay of 80 ns (see the Supporting Information Figure S9 for example fits). Thus, obtained temperature dependence of τ_0_ is plotted in Figure 4(c). As the temperature is increased from 16 K, we see that τ_0_ initially strongly shortens from 750 to about 150 ns and then, above ∼100 K, remains approximately constant. Since the PL intensity remains fairly constant in this temperature range (see Supporting Information Figure S10) the acceleration of the PL decay with the temperature is not due to thermal activation of nonradiative processes. Instead, we attribute the shortening of τ_0_ with increasing temperature to a consequence of emission from a luminescent state split into a lower energy, long-lived, dark state |*F*⟩ and a higher energy bright state |*A*⟩, see the inset in Figure 4(c). The fine structure splitting of the luminescent state, Δ*E*, occurs as a result of electron–hole exchange interaction, and its influence on PL lifetimes has been observed for many binary II–VI colloidal QDs,^57−59^ lead halide perovskites,^60^ CuInS_2_ QDs,^30,32^ and stoichiometric AgInS_2_.^61^ As the temperature is increased, the thermalization between the split states leads to an increased occupation probability of |*A*⟩ and a shorter PL lifetime. To model the temperature dependence of τ_0_, we assume that electron and hole states are pure spin states; i.e., the total angular momentum of the electron and the hole are both equal to 1/2. Note that the assumption of complete quenching of the hole orbital momentum is expected in the case when the hole localization energy exceeds the spin–orbit interaction.^62,63^ As a result of the exchange coupling, the luminescent state is split into a singlet state (*S* = 0) and a triplet state (*S* = 1). Because of the spin selection rule (Δ*S* = 0), the emission from the singlet is optically allowed, and the emission from the triplet is forbidden. The resulting temperature dependence of the PL lifetime is given by^26,30^
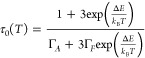
5where Γ_*A*,*F*_ are the radiative recombination rates from |*A*⟩ (singlet) and |*F*⟩ (triplet) and the factor of 3 results from the triplet-to-singlet degeneracy ratio. Fitting of eq 5 yields Δ*E* = 6.5 meV. This value lies roughly in the range of the reported fine structure splittings reported for CuInS_2_, AgInS_2_, CdSe, and InP QDs.^30,57,58,61^ On the other hand, the reported exchange splittings for electron–hole pairs occupying emissive donor–acceptor pairs are smaller than 1 meV for nearest neighbor pairs and quickly decrease with donor–acceptor distance.^64^ In other words, for a DAP luminescent state, we expect Δ*E* to be smaller by almost an order of magnitude.

So far we have presented experimental evidence that in the case of QDs in sample A1 the PL mechanism is not governed by DAP, but by a recombination of a delocalized electron with a localized hole. Let us now consider under what circumstances DAP mechanism can be responsible for PL of *any* QD material. As shown experimentally and theoretically by Ekimov et al., when the QD diameter is sufficiently small, the quantization energy exceeds the interaction between the electron and the ionized donor.^65^ As a result, the electron spontaneously occupies the quantized delocalized state. The critical QD radius below which this autoionization occurs is given by *a*_*C*_ ≈ 2.5*a*_*B*_, where *a*_*B*_ is the Bohr radius of the lowest orbit of the hydrogen-like donor.^65^ The value of *a*_*B*_ is related to the donor binding energy *E*_*B*_ via , where *m*_*e*_ is the electron effective mass. Therefore, the DAP mechanism can be responsible for the PL only if the donor binding potential is sufficiently deep to overcome the quantum size effect. In AgInS_2_, the largest reported donor binding energies are ∼100 meV,^17,66^ which corresponds to a *a*_*C*_ ≈ 4.0 nm. Clearly, for sample A1, with a radius of ∼1.9 nm, DAP recombination is not expected.

Let us now discuss the results obtained on sample A2 containing rod-shaped QDs. In this case, the mean radius of ∼2 nm is smaller, while the mean length of 9 nm is larger than *a*_*C*_ estimated above. Despite the different morphologies, we find that the room temperature TA and PL dynamics are quantitatively similar for samples A1 and A2 (compare Figures 2 and S3). Crucially, in A2 as in A1, XB decay is not accompanied by the rise in PL (see Figure S3(e)) indicating that delocalized electrons participate in the PL process. Moreover, room temperature PL transients and temperature dependence of PL transients measured for A2 and plotted in Figure 4(e) and 4(f), respectively, are quantitatively similar to the respective transients for A1 (Figure 4(b) and 4(c)). These comparisons indicate that in sample A2, as in sample A1, the PL results from the recombination of a delocalized electron with a localized hole.

To evaluate τ_0_, we derive analogues of eqs 3 and 4 taking into account the rod shape (see the Supporting Information, Section S10). We find that the model curve fits the measured PL transients well, see Figure S11. The temperature dependence of τ_0_ together with a fit using eq 5 is shown in Figure S12. The obtained bright-dark splitting is 11.0 meV, i.e., almost twice that for sample A1. However, the volume of QDs in A2 is on average 16 times larger than the QD volume in A1. We therefore expected a smaller splitting for A2 than for A1 because of a strongly reduced spatial overlap of electron and hole wave functions.^67,68^ To explain this discrepancy, we recall that weaker quantization along the nanorod long axis promotes strong electronic correlations.^69^ As a consequence, the ground electron–hole pair state contains admixtures of higher electron states. Since the hole is localized, the correlations lead to squeezing of the pair wave function along the long axis compared to the electron wave function. In other words, the effective volume occupied by the pair is smaller than the nanorod volume. A similar, albeit weaker, effect of electronic correlations in CuInS_2_ QDs was explored in ref (29). Since detailed accounting for correlations is beyond the scope of the present work, we estimate τ_0_ using a simplified approach. We assume that the electron wave function becomes shrunk to a sphere with a radius equal to the nanorod radius. This assumption may overestimate the effect of correlations but allows us to use eqs 3 and 4 to extract τ_0_ by fitting the PL transients. The result for the room temperature PL transient is shown in Figure 4(d) and example fits for other temperatures are shown in Figure S13. The temperature dependence of τ_0_ together with a fit using eq 5 is plotted in Figure 4(f). The evaluated bright-dark splitting is 8.7 meV. This value is also larger than that for sample A1, but now we can attribute it to the correlation-driven shrinkage of the excited state wave function. Overall, the analysis of PL dynamics presented above supports the conclusion that although the length of the nanorods in sample A2 exceeds *a*_*C*_, the PL is due to a recombination of a delocalized electron with a localized hole.

In conclusion, we have presented experimental evidence that the band edge bleach visible in the TA spectra of spherical and rod-shaped alloyed Ag–In–Zn–S QDs results from electrons occupying the lowest quantized state, delocalized over the QD. The lifetime of this population exceeds the PL rise time by more than an order of magnitude. These observations imply that DAP recombination is not the origin of the PL emission. Moreover, we show that a photoinduced absorption signal is consistent with an intragap transition involving localized holes. As a result, we conclude that the PL occurs as a recombination between delocalized electrons and localized holes, i.e., a free-to-bound mechanism. This conclusion is further supported by the temperature dependence of the PL dynamics and theoretical considerations. These results should enable routes toward further control of light emission from alloyed QDs and inspire new applications.

## Supporting Information Available

Details of experimental methods, results of structural characterization, room temperature absorption, PL, and TA studies on sample A2, excitation power dependence of TA spectra on sample A1, demonstration of carrier cooling, 2D maps of TA spectra, analysis of alternative fitting of room temperature PL transients, example fits of PL transients for various temperatures, temperature dependence of the PL intensity, and analysis of the temperature dependence of PL dynamics on sample A2 (PDF)
Transparent Peer Review report available (PDF)
