# Supplementary material for: Carrier Dynamics and Recombination Pathways in Ag–In–Zn–S Quantum Dots
Source: J Phys Chem Lett. 2024 Oct 11;15(42):10479–87. doi: 10.1021/acs.jpclett.4c02126 (PMC11514015; doi:10.1021/acs.jpclett.4c02126)
Supplement: Supplementary file 1 — jz4c02126_si_001.pdf [file jz4c02126_si_001.pdf]

Supporting Information for Carrier Dynamics and Recombination Pathways in Ag-In-Zn-S Quantum Dots

Adam Ćwilich,[†] Daria Larowska-Zarych,[‡,‖] Patrycja Kowalik,[¶,‡,‖] Kamil Polok,[‡] Piotr Bujak,[¶] Magdalena Duda,[†] Tomasz Kazimierczuk,[§] Wojciech Gadomski,[‡] Adam Pron,[¶] and Łukasz Kłopotowski[*,†]

†*Institute of Physics, Polish Academy of Sciences, 02-668 Warsaw, Poland*

‡*Faculty of Chemistry, University of Warsaw, 02-089 Warsaw, Poland*

¶*Faculty of Chemistry, Warsaw University of Technology, 00-664 Warsaw, Poland*

§*Faculty of Physics, University of Warsaw, 02-093 Warsaw, Poland*

‖*Present address: Institute of Physical Chemistry, Polish Academy of Science, 01-224 Warsaw, Poland*

E-mail: lukasz.klopotowski@ifpan.edu.pl

S1 Materials and Methods

S1.1 Materials

Silver nitrate (99%), indium(II) chloride (99%), zinc stearate (technical grade), 1-dodecanethiol (DDT, 98%), sulfur (99%), 1-octadecene (ODE, 90%), oleylamine (OLA, 70%), methyl viologen dichloride hydrate, 98% were supplied by Sigma-Aldrich (Merck KGaA, Darmstadt, Germany).

S1.2 Synthesis of Ag-In-Zn-S Quantum Dots

All operations were carried out under a constant dry argon flow. Silver nitrate (0.03 g, 0.18 mmol), indium(II) chloride (0.11 g, 0.59 mmol), DDT (0.20 g, 1.0 mmol), and zinc stearate (0.33 g, 0.54 mmol for A1 or 0.66 g, 1.08 mmol for A2) were mixed with ODE (15 mL) in a three-neck flask. The mixture was heated to 150 °C until a homogeneous solution was formed. Then, 1 mL of the S/OLA precursor was quickly injected into the reaction solution. The temperature was increased to 180 °C, and the mixture was kept at this temperature for 60 min. After the mixture was cooled to room temperature, toluene (10 mL) was added, and the reaction mixture was centrifuged; the isolated black precipitate consisting of organic waste and agglomerated particles was separated. The supernatant was treated with 30 mL of acetone, leading to the precipitation of the desired fraction of quantum dots. The quantum dots were separated by centrifugation (7000 rpm, 5 min) and then redispersed in nonpolar solvents such as toluene, dichloromethane, chloroform.

S1.3 Conjugating Ag-In-Zn-S Quantum Dots with Methyl Viologen

15 μl of MV^{2+} solution ($\sim$230 mg of MV^{2+} in 1 ml of methanol) was added to 200 μl of concentrated Ag-In-Zn-S chloroform solution. The sample became cloudy, which was attributed to an excess of undissolved MV^{2+}. The solution was sonicated, evaporated and redissolved in 200 μl of chloroform. The resulting solution was passed through a syringe filter

with a pore size of 0.22 μm. As a result, a clear solution of Ag-In-Zn-S QDs with adsorbed MV^{2+} molecules was obtained.

S1.4 Structural Characterization

Elemental analysis was carried out with a multichannel Quantax 400 energy-dispersive X-ray spectroscopy (EDS) system with a 125 eV xFlash detector 5010 (Bruker) using a 15 kV electron beam energy. High-resolution images were acquired by a Tecnai TF20 X-TWIN (Thermo Fisher Scientific) transmission electron microscope operated at 200 kV.

S1.5 Solution-phase Steady State Absorption and Photoluminescence

The absorption spectra were measured using the Agilent Varian Cary 50 UV-visible spectrophotometer. The photoluminescence (PL) measurements were performed in a custom-built setup using a pulsed laser emitting at 405 nm (Picoquant P-C-405B) as the excitation source. The PL signal was measured using an Andor Newton DU-920P CCD camera coupled with an Andor Kymera 328i monochromator.

S1.6 Femtosecond Transient Absorption

The femtosecond transient absorption setup was described previously.[1] Briefly, the femtosecond laser pulses with 800 nm central wavelength were obtained from a 1 kHz Ti:sapphire regenerative amplifier (Coherent Legend Elite) seeded with a Ti:sapphire oscillator (Coherent Micra). Afterwards, pair of pulses was obtained on a beamsplitter plate, where the stronger one was used for generation of 400 nm second harmonic (SH) pump pulse in β-barium borate crystal, while the other passed through optical delay line and was used for white light continuum (WLC) probe pulse generation in a sapphire or a calcium fluoride plate (the latter was used only during measurements with electron scavenger). The intensity of the pump

pulse was adjusted before the sample with a neutral density filter. Part of the probe pulse was used as a reference pulse, that passed through the sample without overlapping with the pump pulse. Both probe and reference pulses were then fed into a monochromator (Princeton Instruments, Acton 2300, 300 gr/mm grating) and detected with a pair of 1024 pixel linear photodiode arrays (Pasher Instruments) in 419.8-696.0 nm range. The pump-probe delay was scanned up to 3600 ps with a variable time step. The polarizations of pump and probe pulses were almost perpendicular and we verified that for the investigated samples the signal did not depend on the relative polarization of the pulses. The measured NPs signals were chirp-corrected using the polynomial fit of chirp in signal measured for pure solvent. In order to calculate pump fluence we measured the $1/e^2$ beam radius (0.32 mm) at the sample using the knife-edge method. The sample concentration was adjusted in order to obtain absorbance of $\sim$ 0.2 in a 2 mm fused silica optical cuvette. The expected full-width at half-maximum (FWHM) of the 400 nm pump pulse, estimated using the pulse spectrum (4.86 nm FWHM) and dispersion of optical elements in the setup, is ~100 fs (~128 fs for measurements with electron scavenger). The width of the instrumental response function (IRF) ranged from ~360 fs at 480 nm to ~640 fs at 696 nm for WLC generated in sapphire and from ~240 fs at 426 nm to ~580 fs at 696 nm for WLC generated in calcium fluoride. IRF width was estimated from optical Kerr effect (OKE) signal measurement for pure solvent. In the OKE configuration, where the optically induced birefringence was measured, a polarizer was placed on the probe pulse path after the sample at right angle with respect to the probe pulse polarization, while the polarization of the pump pulse was changed to 45° with respect to the probe pulse.

S1.7 Temperature-dependent Photoluminescence

For measurements of PL dynamics as a function of the temperature a drop of sample solution was spin-coated (1500 rpm) onto a clean silicon oxide wafer. The sample was mounted on a variable temperature insert inside a split-coil cryostat (Cryomag, Oxford Instruments), i.e.,

in helium exchange gas. The PL signal was excited using a pulsed laser (Hamamatsu C8898) operating at a repetition frequency of 10 kHz. The PL signal was collected using a lens placed outside the cryostat and directed onto the an avalanche photodiode (APD, Excelitas SPCMAQRH-15). The PL decays were histogrammed using a time-correlated single photon counting unit (Picoquant HydraHarp 400). The overall temporal resolution was about 1 ns.

Temperature dependence of PL spectra were measured on analogously prepared samples. The wafer with QDs was placed inside a cold-finger cryostat (Microstat Hi-Res, Oxford Instruments) and excited with a pulsed laser emitting at 405 nm (Picoquant P-C-405B). The PL signal was detected using a CCD camera (Andor iDus DV420A-OE) coupled to a monochromator (Andor Shamrock SR500i).

S1.8 Photoluminescence Quantum Yield

The PL quantum yields (QYs) of the samples were determined using the relative method.[2] The PL spectra of the samples and the QY standard (Rhodamine 6G) were measured using a spectrophotometer (Fluorolog, Horiba Jobin-Yvon) with the excitation wavelength of 488 nm. PL QY was calculated as:

$$\mathrm{QY}_x = \mathrm{QY}_{st} \frac{F_x}{F_{st}} \frac{f_{st}}{f_x} \frac{n_x^2}{n_{st}^2}$$

where $\mathrm{QY}_{st} = 0.95$, $F_{st,x}$ are the integrated PL intensities of the standard and the sample, $n_{st,x}$ are the refractive indices of the standard and sample solvents (ethanol and toluene, respectively) at the emission wavelength, and $f_{st,x} = 1 - 10^{-A_{st,x}}$ are the absorption factors with $A_{st,x}$ being the absorbance of, respectively, the standard and sample at the excitation wavelength.

S2 Results of Structural Characterization

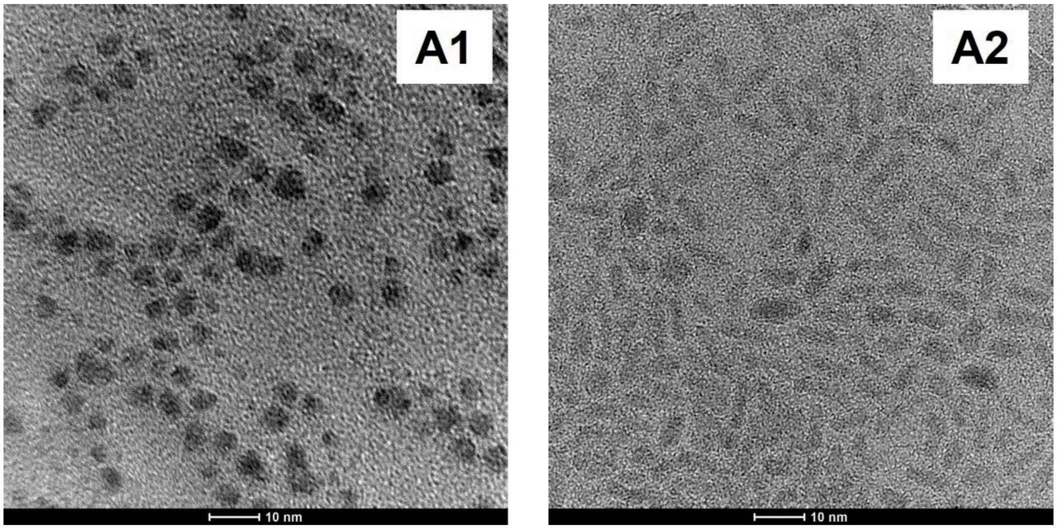

Figure S1: High resolution transmission electron micrographs of sample A1 (left) and A2 (right). The image of A1 reveals spherical QDs and the image of A2 shows rod-shaped nanocrystals. Detailed analysis of size distribution is published in Ref. 3. (Note different labeling of samples in Ref. 3.) The QDs in A2 exhibit a diameter (3.7 ± 0.7) nm and an average aspect ratio of 1.1 ± 0.1. A2 consists of nanorods with an average diameter of (3.9 ± 0.9) nm and an aspect ratio of 2.4 ± 0.7.

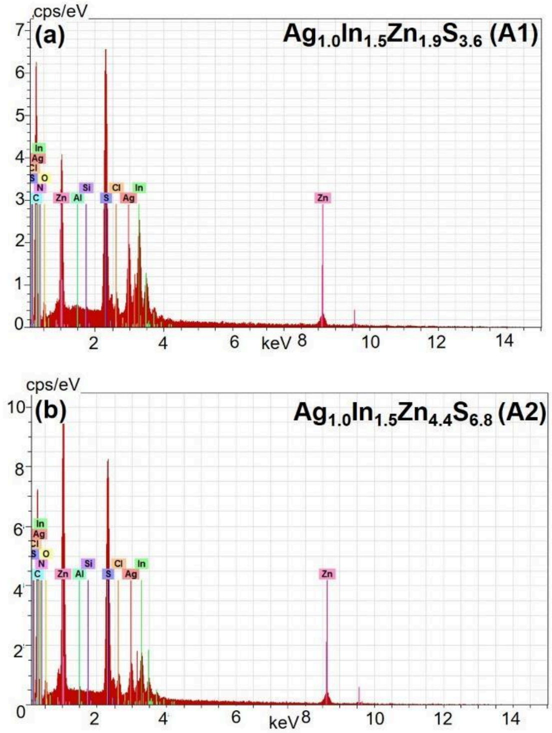

Element	Series	unn. C [wt.%]	norm. C [wt.%]	Atom. C [at.%]	Error (1 Sigma) [wt.%]
Carbon	K-series	44.03	45.83	73.37	5.64
Nitrogen	K-series	3.29	3.43	4.70	0.87
Oxygen	K-series	7.02	7.31	8.78	1.28
Aluminium	K-series	0.04	0.04	0.03	0.03
Silicon	K-series	0.06	0.06	0.04	0.03
Sulfur	K-series	9.19	9.57	5.74	0.35
Chlorine	K-series	0.73	0.76	0.41	0.05
Zinc	K-series	9.84	10.24	3.01	0.41
Silver	L-series	8.45	8.79	1.57	0.30
Indium	L-series	13.43	13.98	2.34	0.44
	Total:	96.09	100.00	100.00	

Element	Series	unn. C [wt.%]	norm. C [wt.%]	Atom. C [at.%]	Error (1 Sigma) [wt.%]
Carbon	K-series	50.22	50.31	72.80	6.44
Nitrogen	K-series	4.58	4.59	5.69	1.13
Oxygen	K-series	9.65	9.67	10.50	1.64
Aluminium	K-series	0.07	0.07	0.04	0.03
Silicon	K-series	0.02	0.02	0.01	0.03
Sulfur	K-series	9.53	9.54	5.17	0.37
Chlorine	K-series	1.18	1.18	0.58	0.07
Zinc	K-series	12.56	12.59	3.34	0.51
Silver	L-series	4.70	4.70	0.76	0.18
Indium	L-series	7.33	7.34	1.11	0.26
	Total:	99.82	100.00	100.00	

Figure S2: Energy-dispersive spectra of sample A1 (a) and sample A2 (b) together with elemental analysis.

S3 Room Temperature Studies for Sample A2

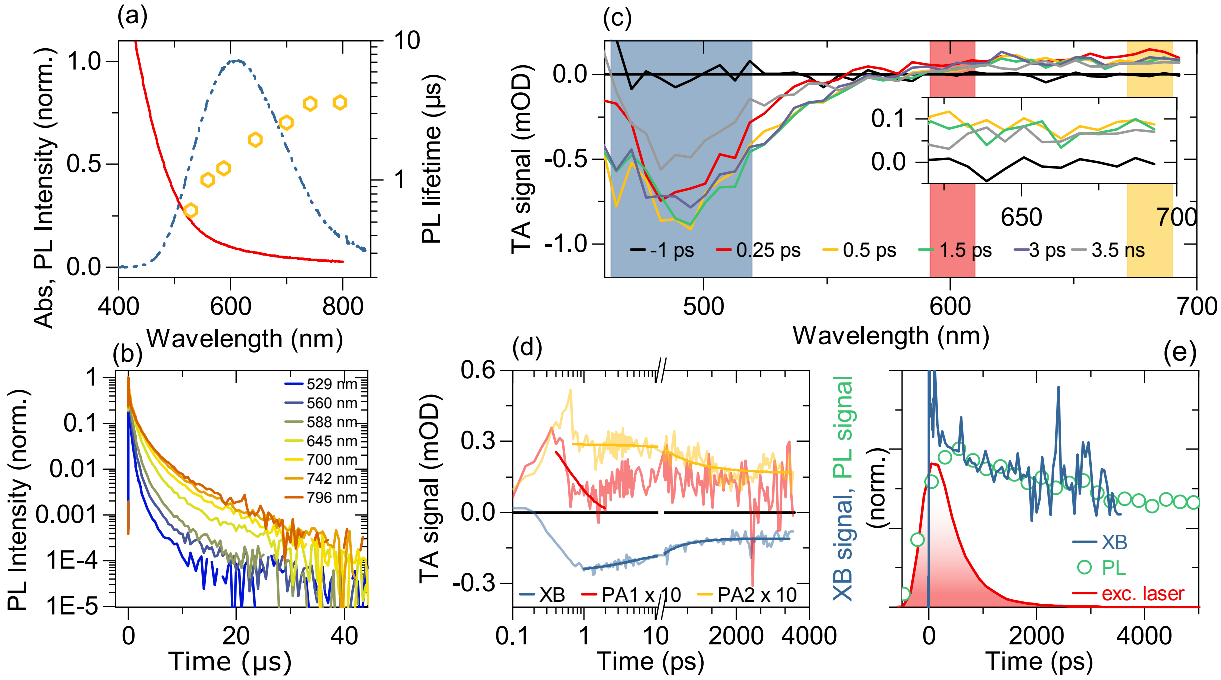

Figure S3: (a) Absorption (solid line) and PL (broken line) spectra of sample A2. Yellow hexagons denote detection wavelength dependence of average PL lifetimes. (b) Normalized PL decays of sample A2 as for various detection wavelengths given in the legend. (c) Transient absorption spectra for sample A2 for various pump-probe delays indicated in the legend. The inset shows a magnification of the red edge of the spectra. (d) TA kinetics for three signals denoted in (c): ground state bleach (XB, blue) and two photoinduced absorption bands (PA1 – red and PA2 – yellow). Thick semitransparent lines denote experimental transients, thin lines denote fits of eq. 2 of the main text. (e) Comparison of TA kinetics (blue line) and PL dynamics (green open points). Red shaded area denotes the temporal profile of the laser used for PL excitation.

S4 Pump-power-dependent Transient Absorption

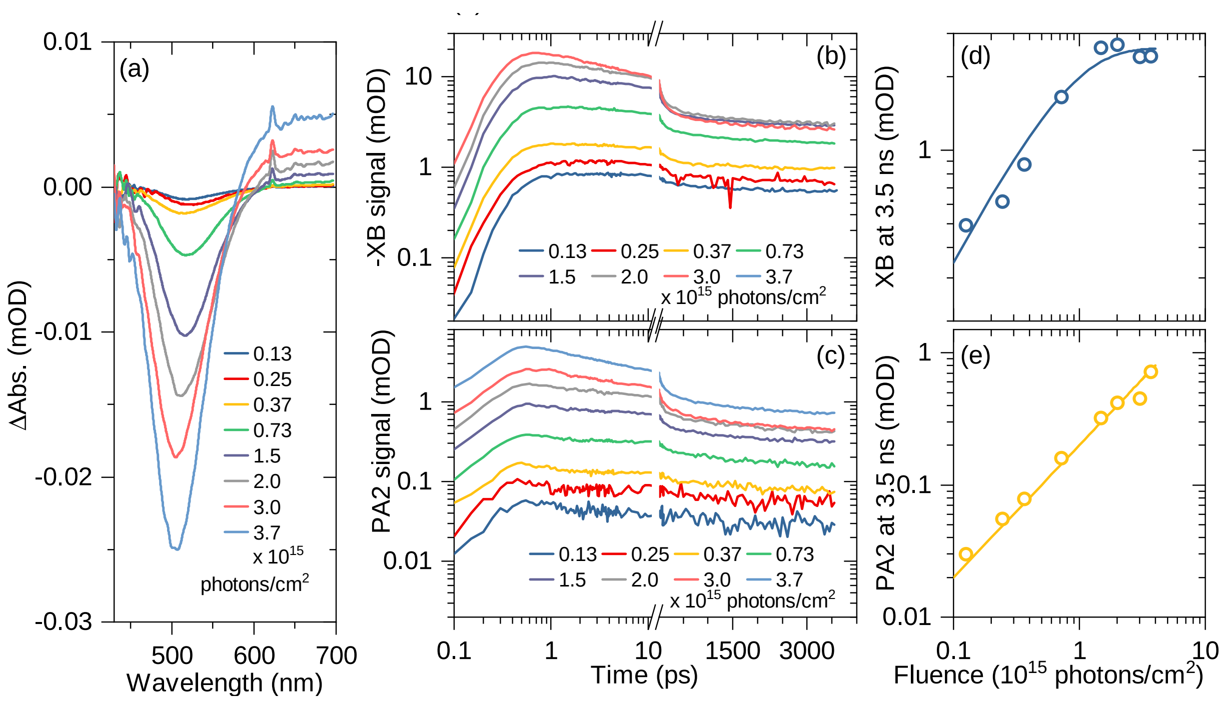

Figure S4: (a) Fluence dependence of the TA spectra at pump-probe delay times where maximum ground state bleach is attained. (b) TA kinetics of ground state bleach, XB. (c) Ta kinetics of photoinduced absorption, PA2. The signals in (b) and (c) are plotted in double logarithmic scale. Note that the values of the XB signal are inverted. (d) Fluence dependence of the XB signal at 3.5 ns. Points denote experimental data and line is a fit of $\mathrm{XB}(3.5\text{ ns}) = C(1 - \exp(-\sigma J))$, where J denotes the fluence in photons/cm^2 and σ denotes the absorption cross section. Note the saturation of XB(3.5 ns) with increasing fluence. Importantly, the fit yields $\sigma = 1.42 \times 10^{-15}$ cm ^{2}. The fit allows to evaluate the average number of photoexcited electron hole pairs as $\langle N_0 \rangle = \sigma \cdot J$. (e) Fluence dependence of the PA2 signal at 3.5 ns. Points denote experimental data and line is a linear fit, i.e., $\mathrm{PA2}(3.5\text{ ns}) = D \cdot J$, where D is a constant.

S5 Carrier Cooling

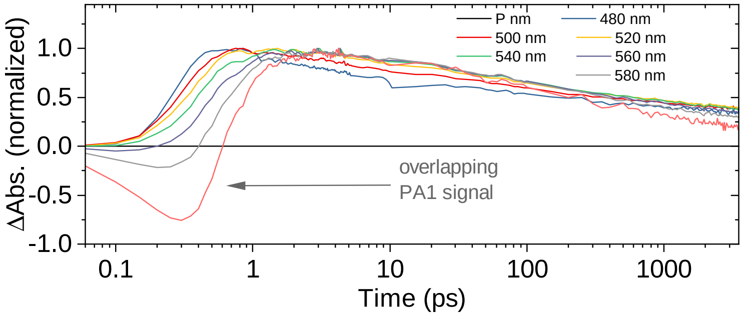

Figure S5: TA kinetics for various wavelengths across the ground state bleach signal. Note that the bleach develops faster at shorter wavelengths than at longer wavelengths as discussed in the main text. We attribute this spectral dependence to electron cooling. The negative signal visible a high fluences and short pump-probe delays is related to an overlap between the ground state bleach (XB signal) and the short lived photoinduced absorption (PA1 signal). Excitation fluence for this data is 7.3×10^{14} photons/cm^2.

S6 2D Maps of Transient Absorption Spectra

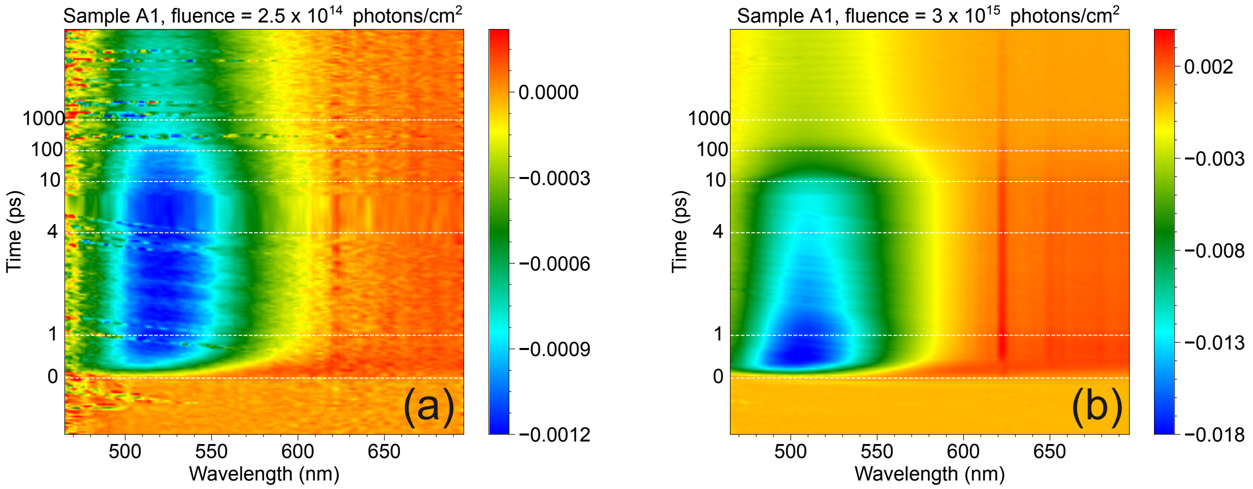

Figure S6: Two dimensional TA maps for sample A1 and two excitation fluences: (a) 2.5×10^{14} photons/cm^2 and (b) 3×10^{16} photons/cm^2. Note the short-lived and long-lived photoinduced absorption components. The feature visible at ~ 625 nm is an artifact.

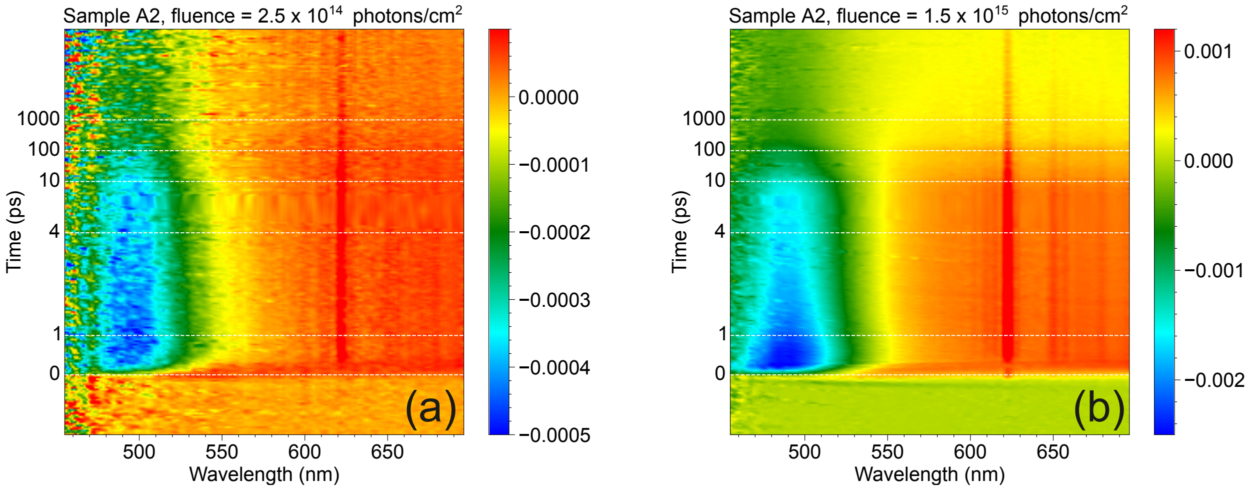

Figure S7: Two dimensional TA maps for sample A2 and two excitation fluences: (a) 2.5×10^{14} photons/cm^2 and (b) 1.5×10^{15} photons/cm^2. Note the short-lived and long-lived photoinduced absorption components. The feature visible at ~ 625 nm is an artifact.

S7 Alternative fitting of PL dynamics of sample A1

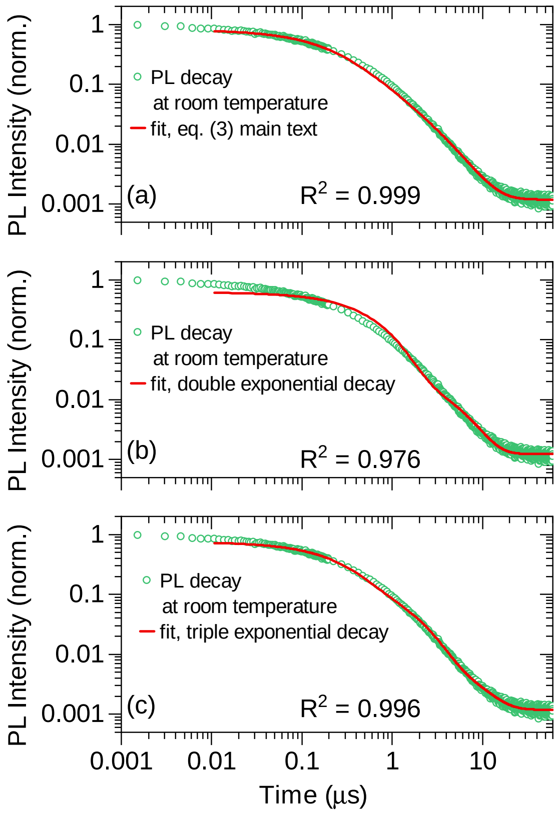

Figure S8: Comparison of different fitting results of the room temperaure PL transient. In all panels open points denote the experimentally measured transient. (a) Line: fit of the model incorporating homogeneous distribution of hole localization sites, eq. 3 of the main text. This is a replot of Fig. 4(a) of the main text for comparison. (b) Line: fit of a double exponential decay function. (c) Line: fit of triple exponential decay function. The number of fitting model parameters is 3 in (a), 5 in (b) and 7 in (c). The goodness of fit measured with reduced r-squared test shows that the best fit is achieved in (a).

S8 Fitting of temperature dependent PL dynamics of sample A1

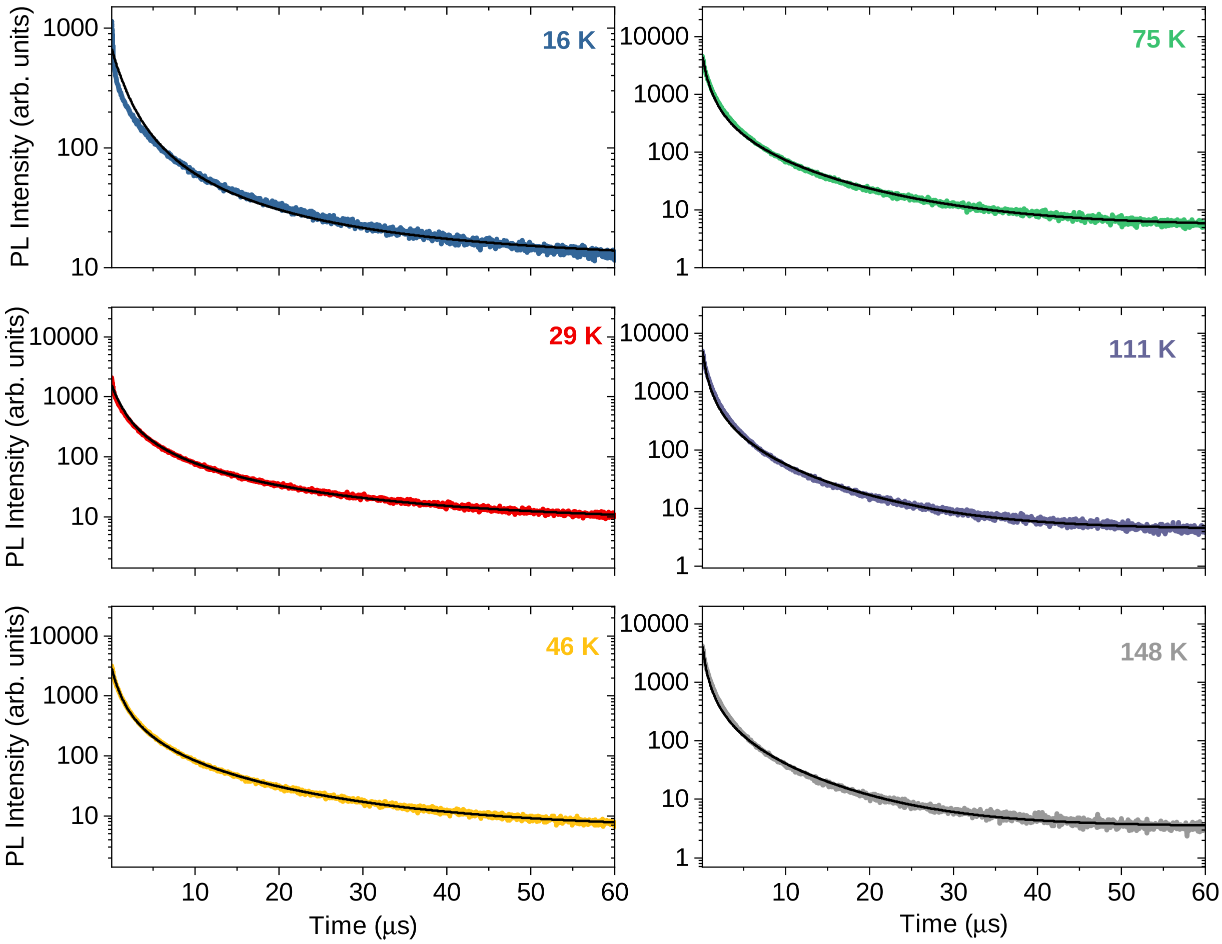

Figure S9: Example fits of PL transients for various temperatures. Color thick lines denote experimental data. Thin black lines denote fits of eq. 3 of the main text.

S9 Temperature dependence of photoluminescence intensity

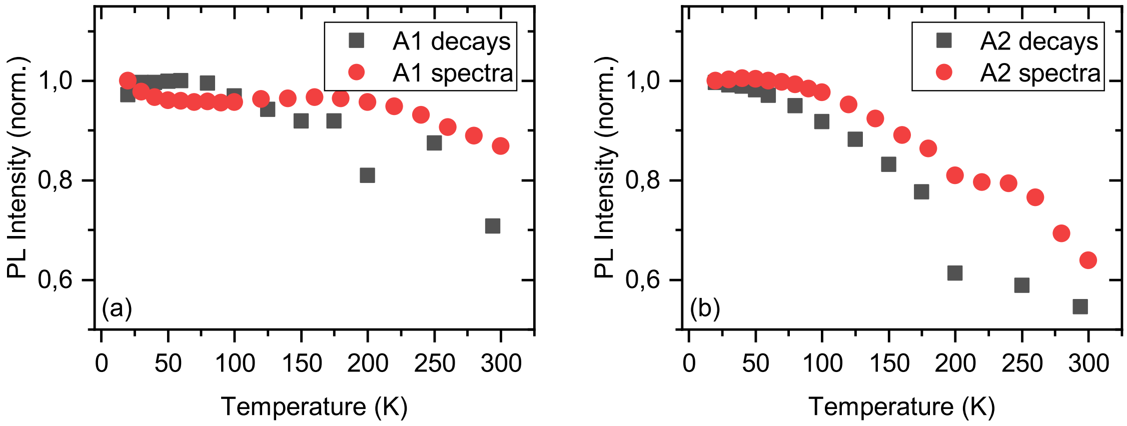

Figure S10: Temperature dependence of PL intensities for sample (a) A1 and (b) A2. The intesities are obtained by temporal integration of PL decays (squares) and spectral integration of PL spectra (circles). Note that in the range, where the PL lifetime changes rapidly, between 5 and 100 K, the PL intensity changes by less than 10%. We therefore conclude that the strong changes in the lifetime are not due to activation of nonradiative channels. Above 150 K (100 K) for sample A1 (A2), the PL intensity starts to drop indicating increased nonradiative decay rate. However, in this temperature range, the change in lifetime is small and completely accounted for by our equilibrium formula (eq. 5 of the main text). From these observations, we conclude that the activation of non-radiative recombination does not lead to a decreased PL lifetime. This in turn remains in agreement with results of Mooney et al.,[4] which indicated that increasing the temperature leads to an increasing large fraction of non-emissive QDs. Note also that the temperature behaviors of the PL intensities for A1 and A2 are consistent with different PL QYs for these samples: the thermal activation of the nonradiative emission for A2 is stronger than for A1 while the PL QY of A2 is somewhat smaller than of A1.

S10 Photoluminescence dynamics of sample A2

Our initial approach of fitting the PL decays for the A2 sample containing rod-shaped nanocrystals was to derive an analog of the model for ensemble PL dynamics for spherical QDs.[5] To this end, we treated the nanorod shape as a cylinder with a radius $a = 3.9$ nm and length $L = 9$ nm. We solved the Schrödinger equation in cylindrical coordinates for an electron confined to such a cylinder by a potential which is zero inside and infinity outside of it, i.e.,

$$V(\rho, \phi, z) = \begin{cases} 0, & \text{if } 0 \leq \rho < a \text{ and } 0 \leq z \leq L, \\ \infty, & \text{otherwise.} \end{cases} \tag{S1}$$

The solution for the ground state electron wavefunction is

$$\psi_e(\rho, \phi, z) = \frac{1}{\sqrt{\pi} a J_1(\alpha_{0,1})} \sqrt{\frac{2}{L}} \cos\left(\frac{\pi z}{L}\right) J_0\left(\frac{\alpha_{0,1}}{a}\rho\right) \tag{S2}$$

where J_m is an mth order Bessel function of the first kind, and $\alpha_{0,1}$ is the 1st zero of J_0.

To evaluate the distribution of PL lifetimes, we assume that hole localization sites are distributed uniformly inside the volume of a nanorod with a radius $\rho_{\max} < a$ and length $L_{\max} < L$. As noted in the main text, such an approach assumes negligible recombination probability for sites close to the surface. The radiative lifetime τ is proportional to the square overlap of the electron wavefunction given by eq. S2 and the hole wavefunction given by $\psi_h(\mathbf{r}) = \delta(\mathbf{r} - \mathbf{r}_{\text{loc}})$:

$$\tau(\rho_{\text{loc}}, z_{\text{loc}}) = \tau_0 \left(J_0\left(\frac{\alpha_{0,1}\rho_{\text{loc}}}{a}\right) \cos\left(\frac{\pi z_{\text{loc}}}{L}\right)\right)^{-2} \tag{S3}$$

We then fit the measured PL transients with

$$I(t, A, \tau_0, \rho_{\max}, z_{\max}, I_0) = A \int_0^{\rho_{\max}} \int_{-L_{\max}/2}^{L_{\max}/2} \frac{\exp(-t/\tau(\rho_{\text{loc}}, z_{\text{loc}}))}{\tau(\rho_{\text{loc}}, z_{\text{loc}})} 2\pi\rho_{\text{loc}} dz_{\text{loc}} d\rho_{\text{loc}} + I_0. \tag{S4}$$

Example fits for $\rho_{\max} = 3.5$ nm and $L_{\max} = 8.6$ nm are shown in Fig. S11.

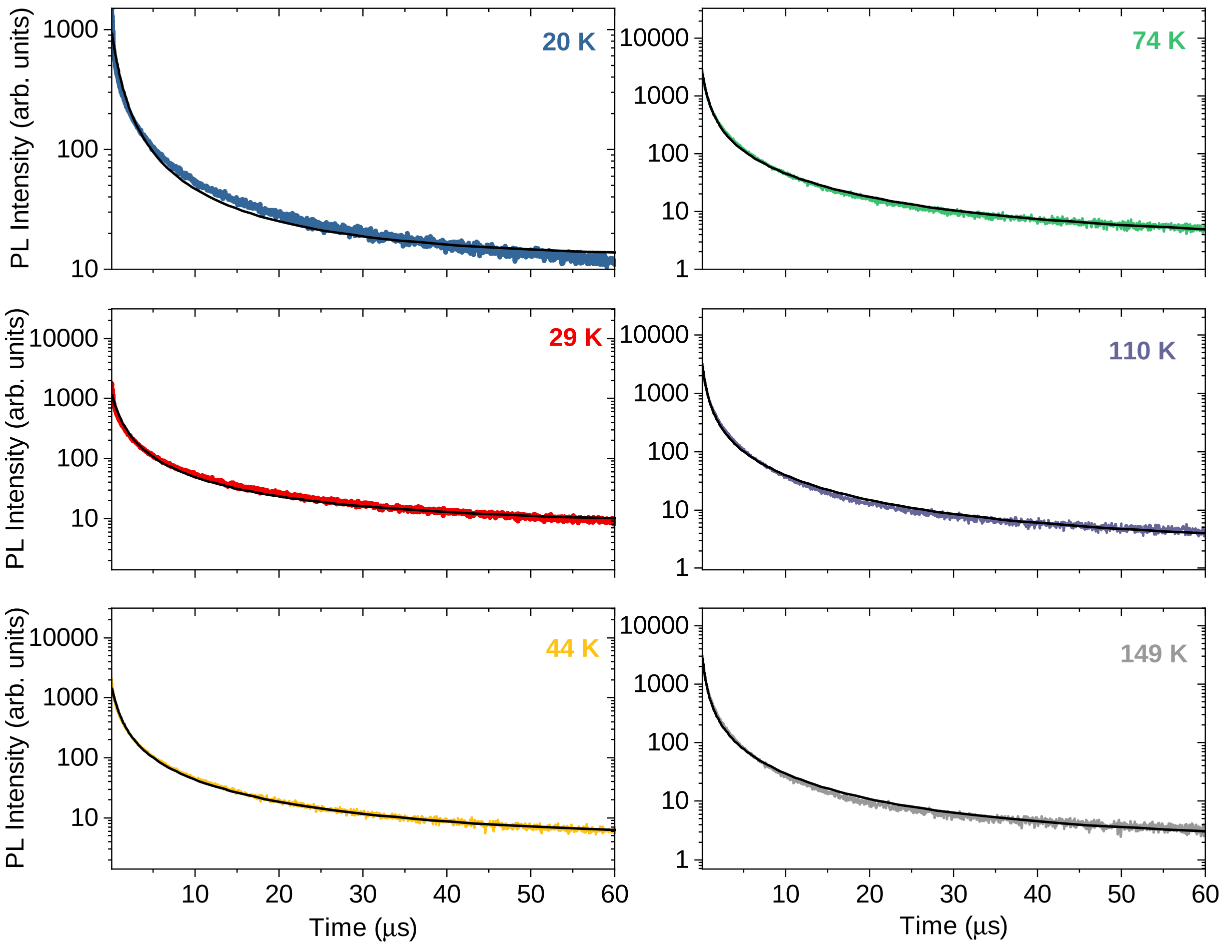

Figure S11: Example fits of PL transients for sample A3 and various temperatures. Color thick lines denote experimental data. Thin black lines denote fits using the nanorod model, i.e., eq. S4

The obtained values of τ_0 are plotted as a function of temperature as open points in Fig. S12. The line indicates a fit with eq. 5 of the main text. This fit allows us to evaluate the bright-dark splitting ΔE. For the values obtained using the nanorod model (eq. S4), we get $\Delta E = 11.0 \pm 0.2$ meV. As we argue in the main text, this value is inconsistent with the nanorod model, since we expected ΔE to be smaller than for the spherical QDs in sample A1 which have a smaller volume. In the main text, we attribute this discrepancy to electronic correlations.

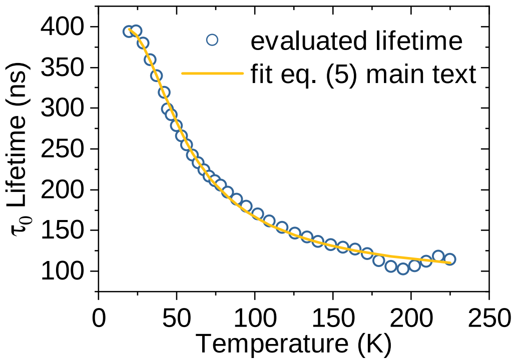

Figure S12: Points: τ_0 lifetimes evaluated from the PL decays by fitting the nanorod model (eq. S4). Line: fit with eq. 5 of the main text.

To estimate τ_0 influenced by electron correlations, we fit the experimental PL transients with the function given by eq. 3 of the main text. Example fits are plotted in Fig. S13.

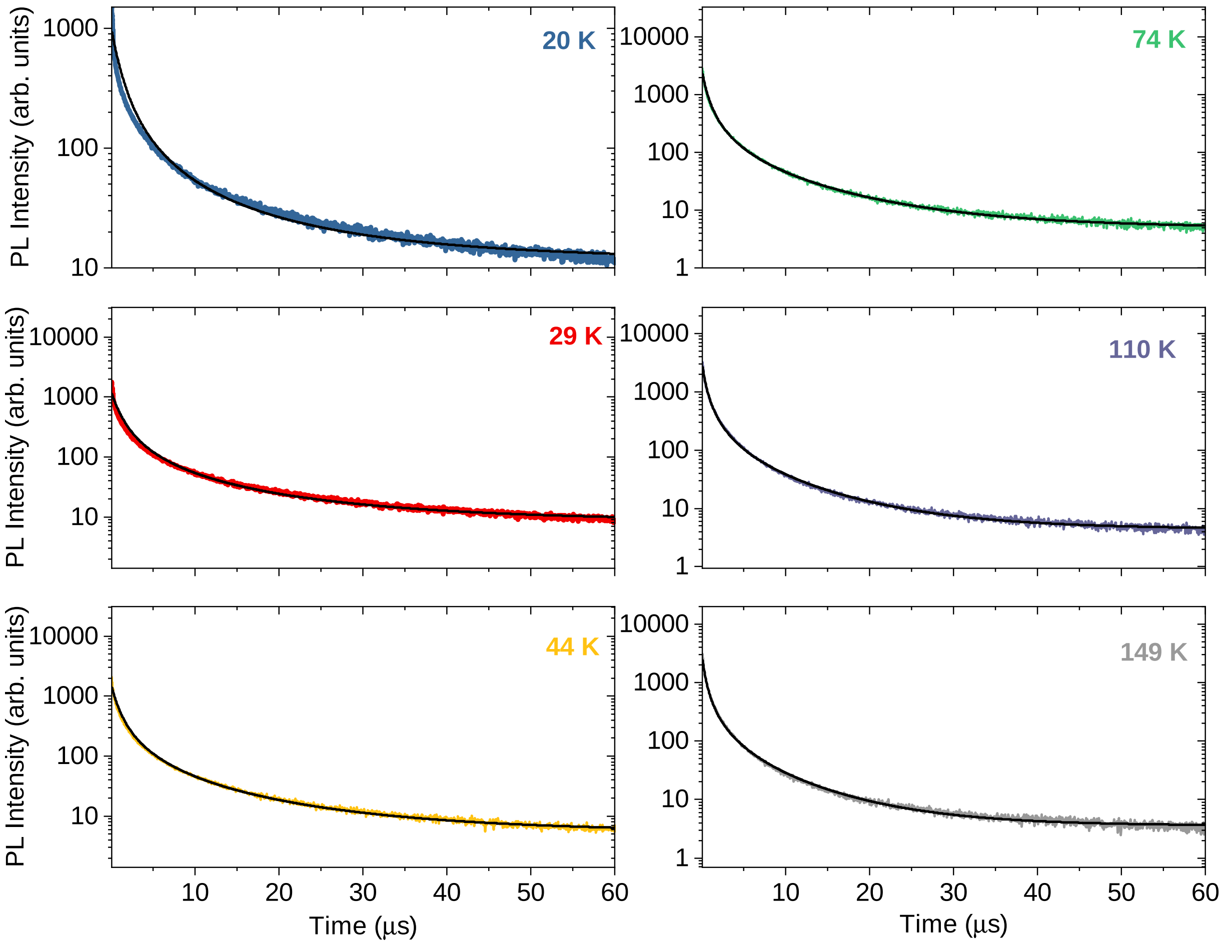

Figure S13: Example fits of PL transients for sample A3 and various temperatures. Color thick lines denote experimental data. Thin black lines denote fits using the spherical QD model, i.e., eq. 3 of the main text.

References

(1) Piątkowski, P.; Ratajska-Gadomska, B.; Gadomski, W. Probing slow dynamics by ultrafast process: Sol–gel transition detected by transient absorption spectroscopy of quantum dots. *Journal of Molecular Liquids* **2012**, *176*, 106–111.

(2) Würth, C.; Grabolle, M.; Pauli, J.; Spieles, M.; Resch-Genger, U. Relative and absolute determination of fluorescence quantum yields of transparent samples. *Nature protocols* **2013**, *8*, 1535–1550.

(3) Kowalik, P.; Bujak, P.; Penkala, M.; Maroǹ, A. M.; Ostrowski, A.; Kmita, A.; Gajewska, M.; Lisowski, W.; Sobczak, J. W.; Pron, A. Indium (II) Chloride as a Precursor in the Synthesis of Ternary (Ag–In–S) and Quaternary (Ag–In–Zn–S) Nanocrystals. *Chemistry of Materials* **2022**, *34*, 809–825.

(4) Mooney, J.; Krause, M. M.; Saari, J. I.; Kambhampati, P. A microscopic picture of surface charge trapping in semiconductor nanocrystals. *The Journal of Chemical Physics* **2013**, *138*, 204705.

(5) Hinterding, S. O.; Mangnus, M. J.; Prins, P. T.; Jöbsis, H. J.; Busatto, S.; Vanmaekelbergh, D.; de Mello Donega, C.; Rabouw, F. T. Unusual Spectral Diffusion of Single $CuInS_2$ Quantum Dots Sheds Light on the Mechanism of Radiative Decay. *Nano Lett.* **2021**, *21*, 658–665.
