# Supplementary material for: Carrier Dynamics and Recombination Pathways in Ag–In–Zn–S Quantum Dots
Source: J Phys Chem Lett. 2024 Oct 11;15(42):10479–87. doi: 10.1021/acs.jpclett.4c02126 (PMC11514015; doi:10.1021/acs.jpclett.4c02126)
Supplement: Supplementary file 2 — jz4c02126_si_002.pdf [file jz4c02126_si_002.pdf]

jz-2024-02126w.R1

Name: Peer Review Information for "Carrier Dynamics and Recombination Pathways in Ag-In-Zn-S Quantum Dots"

First Round of Reviewer Comments

Reviewer: 1

Comments to the Author

Reviewer's remarks on the manuscript “Carrier Dynamics and Recombination Pathways in Ag-In-ZnS Quantum Dots”

The manuscript presents a comprehensive and methodical study of photophysical processes in alloyed non-stoichiometric Zn-Ag-In-S (ZAIS) quantum dots (QDs) by combining transient absorption (TA) with and without an electron donor (MV2+) and photoluminescence (PL) decay studies at different temperatures T. The authors show that the conventional donor-acceptor pair (DAP) PL mechanism cannot satisfactorily explain the experimental observations, at least, for the sizes the authors have under the present study. The results are reliable and consistently discussed. The paper is important and interesting for the QD community of The Journal of Physical Chemistry.

The paper undoubtedly deserves to be published. Some minor critical comments (see below) can be accounted for by a minor revision, which is recommended for the manuscript prior the publication.

1. QD size distribution is not calculated from TEM data and it is not taken into account in the discussions of the TA dynamics. Definitively, it can contribute to the inhomogeneous broadening, dynamics, and redshift of the TA signal as well as the electron cooling, considering the size range of the present QDs.

2. Authors conclude that XB originates from delocalized electrons, It would be good to have some explanation for the bleaching - Burstein-Moss effect?

3. Where MV+cation-radical disappears after 3.5 ns? Is oxygen present in the system to capture the electron or the electron is transferred back to QD? Please comment.

4. The authors mention that PL intensity does not noticeably change in the studied T window. It is somehow in contradiction with previous reports showing a strong increase in the PL intensity of AIS-based QDs with lowering T (at least between RT and 100-150 K). At the same time, no data can be found in the paper and SI on the evolution of the intensity and spectral parameters of PL with temperature to make independent conclusions.

5. The authors dismiss the DAP model and suggest a mechanism explaining PL as a result of the radiative recombination between delocalized electron and trapped hole. Please comment on the origin of the large spectral width of the PL band in the frame of this model.

6. No clear reasons are provided for the sample A2? Was it used to compare nanorods with QDs? So, what is the outcome? Are the identical? Then A2 becomes redundant.

Reviewer: 2

Comments to the Author

The authors prepared the two types of Ag-In-Zn-S QDs separately with spherical shape (sample A1) and rod shape (sample A2), and these samples were analyzed by transient absorption spectroscopy. Contrary to the conventional understanding that the broad emission of Ag-In-Zn-S QDs is due to D-A pairs, the results suggest the possibility that delocalized electrons and localized holes are responsible for the majority of the emission. From the results of temperature-dependent PL decay measurements, the relationships between the spatial distribution and energy of the acceptor levels within the particles have been considered.

Overall, the analysis and discussion are detailed, providing some new insights, and the reviewer considers the paper to be acceptable for publication in J. Phys. Chem. Lett. However, the mention of sample A2 is mostly limited to the supporting information, with references to it “being analogous to sample A1” in the main text, making its significance unclear. Recently, band-edge emission by shell formation of group 13-16 semiconductors has been reported, and it has been shown that the shape and compositional distribution within particles significantly affect the band structure and defect levels. If there are optical differences between samples A1 and A2 other than shape and composition, they should be clearly stated in the main text.

In addition, please revise the manuscript with particular attention to the following points:

The authors consider that the samples are cation-rich by assuming charge neutrality. However, is there a possibility of oxygen (oxides)? How was the composition determined?

In eq.1, should the last term (Coulomb interaction) be a negative value? Hence, the reviewer considers that "the decays at, respectively, high and low energies, probe donor-acceptor pairs closer and further apart" is opposite.

In Figure 2c, the TA spectrum has been color-coded in certain wavelength ranges, but in my printing environment, the spectrum is obscured (totally veiled). It seems that the image is provided as a metafile, but to avoid this issue, it might be better to provide it as an image file. Additionally, the color scheme of the graph is not very easy to see.

In the regions PA1 and PA2 of Figure 2c, for example, the graph at 0.4 ps does not appear to change by 0.5 mOD. The difference with Figure 2d needs to be explained.

The format of the figure numbers does not match the format of the figure numbers in the text. In the figures, they are written in lowercase letters with parentheses (a), (b), (c), while in the text in some parts they are written as 2A, 2B, 2C in uppercase.

"The presence of the long-lived delocalized carrier population and the lack of a slow rise dynamics in the PL transient rules out the DAP recombination as the PL mechanism." Is the time resolution of PL decay measurement sufficient to distinguish the difference in the lifetime of 36 ns of XB?

Looking at the temperature dependence of the PL decay in Figure 4b, there appear to be at least two lifetime components, one of which appears to become longer with temperature. Since the fitting was performed using eq. 3, which relates to the distance between the quantum dot center and the acceptor site, it appears that the single tau 0 value is shortening (accelerated emission decay, Figure 4c) with temperature. However, is there a component that shows the opposite behavior?

Reviewer: 3

Comments to the Author

This manuscript presents transient absorption and temperature-dependent PL dynamics data of Ag-In-Zn-S nanocrystals that are consistent with a decay mechanism that involves a delocalized quantum-confined electron recombining with a localized hole. This mechanism contrasts with the

alternative donor-acceptor-pair (DAP) mechanism in which both the electron and hole are localized to defect states. The data and analysis are clearly presented with a cohesive argument that clarifies the mechanism of PL decay in quaternary sulfide systems. This manuscript therefore represents an informative addition to the literature on this class of materials. I recommend publication after minor revisions to address the following two points.

1. The introduction of the paper contrasts the DAP mechanism with the free-to-bound mechanism and states that the “prevailing view in the literature is that PL occurring [in Ag-In-Zn-S QDs] as a result of the DAP recombination..”. However, this statement has no specific references associated with it. The introduction could be strengthened by including clearer references to specific reports that favor the DAP mechanism for Ag-In-Zn-S nanocrystals.

2. The paper presents spectral data for two different samples: spherical particles (A1) and rod-shaped particles (A2). All of the data in the main text are for sample A1 and the data for A2 are only included in the supporting information. The only mention of the A2 sample in the main text is that it has analogous room temperature transient absorption spectral properties to A1. The manuscript should elaborate on the significance of the data from sample A2, particularly the temperature dependent PL dynamics. Is the value of the dark-bright splitting obtained from the A2 sample similar to that for the A1 sample? How was the model described by equations 3 and 4 adapted for a rod-shaped particle given that it assumes a spherical distribution of hole localization states?

Author's Response to Peer Review Comments:

Response to Reviewers and a Summary of Changes

First and foremost, we would like to thank all the Reviewers for careful reading of our work and valuable suggestions. We believe that Reviewers' comments allowed us to improve the manuscript in the present version. Below, we provide a point-by-point response to all the questions and suggestions.

Reviewer(s)' Comments to Author:

Reviewer: 1

Recommendation: This paper is publishable subject to minor revisions noted. Further review is not needed.

Comments:
Reviewer's remarks on the manuscript “Carrier Dynamics and Recombination Pathways in Ag-In-ZnS Quantum Dots”

The manuscript presents a comprehensive and methodical study of photophysical processes in alloyed non-stoichiometric Zn-Ag-In-S (ZAIS) quantum dots (QDs) by combining transient absorption (TA) with and without an electron donor (MV2+) and photoluminescence (PL) decay studies at different temperatures T. The authors show that the conventional donor-acceptor pair (DAP) PL mechanism cannot satisfactorily explain the experimental observations, at least, for the sizes the authors have under the present study. The results are reliable and consistently discussed. The paper is important and interesting for the QD community of The Journal of Physical Chemistry.

The paper undoubtedly deserves to be published. Some minor critical comments (see below) can be accounted for by a minor revision, which is recommended for the manuscript prior the publication.

We thank the Reviewer for the positive assessment of our work.

1. QD size distribution is not calculated from TEM data and it is not taken into account in the discussions of the TA dynamics. Definitively, it can contribute to the inhomogeneous broadening, dynamics, and redshift of the TA signal as well as the electron cooling, considering the size range of the present QDs.

The size distribution of the samples is given in Ref. 11. From the analysis of the particular TEM images shown in the Supporting Information, we obtain the following results (mean ± standard deviation):. Sample A1 diameter = (4.0±0.5) nm. Sample A2 diameter = (3.2±0.6), length = (8.2±1.5). Definitely, a more precise estimation is given in Ref. 11 since the analysis is performed on larger sets.

In the revised Supporting Information, we cite this information in the caption to Fig. S1:

The QDs in A2 exhibit a diameter (3.7 ± 0.7) nm and an average aspect ratio of 1.1±0.1. A2 consists of nanorods with an average diameter of (3.9 ± 0.9) nm and an aspect ratio of 2.4± 0.7.

The impact of size distribution on TA dynamics is an interesting point. The Reviewer is absolutely right: the inhomogeneous broadening does contribute to the TA dynamics. However, quantitative accounting for the size distribution is virtually impossible. The broadening results in spectral overlap of absorption transitions – the transitions to excited electron states (2S, 1P, 1D, etc.) overlap with the transitions to the electron ground state (1S). Thus, we cannot distinguish them. As a result, the effect of electron cooling is seen as a transient shift of the XB band to longer wavelengths. We discussed this on page 8. However, the Reviewer raises the issue of a size dependent cooling, which we have not discussed. In analogy to the case of CdSe QDs (see Klimov

et al. Phys. Rev. B 60 13740, 1999), we expect that the cooling rate will increase with decreasing QD radius. This will make the XB at highest energies (reflecting electrons at excited states in small dots) decay faster than XB at less high energies (due to electrons at excited states in large dots). Clearly, we can not separate these contributions because of the overlap with transitions due to ground state electrons. To add this scenario to our discussion, when discussing the transient XB shift, we add on page 8

The ps dynamics of this shift reflects a superposition of size-dependent electron cooling.

2. Authors conclude that XB originates from delocalized electrons, It would be good to have some explanation for the bleaching - Burstein-Moss effect?

The XB signal originates from filling of the electron states. Due to Pauli exclusion principle, the filling leads to blocking of the absorption via these states. To clarify, on page 10, we add Therefore, we associate the XB signal with filling of delocalized electron states with negligible contribution from the hole states.

3. Where MV+cation-radical disappears after 3.5 ns? Is oxygen present in the system to capture the electron or the electron is transferred back to QD? Please comment.

This is a difficult question. Definitely we do not see any effect of back electron transfer (i.e., no reappearance of the ground state bleach). The lack of back electron transfer is in fact expected from the comparison of the energy position of the conduction band minimum (E_{CBM}) of Ag-In-Zn-S QDs with the MV reduction potential. Kameyama et al. (Ref. 23) reported that E_{CBM} lies in the range between -1 and -1.5 V vs NHE for QDs with ~5 nm in diameter and various Ag/Zn compositions. Note that E_{CBM} will become more negative for our QDs with a diameter < 4 nm. On the other hand, the reduction potential of MV is about -0.45 V vs NHE. Thus, the back transfer is energetically unfavorable. However, the Reviewer is right that the photoinduced absorption signal indicating presence of reduced
MV disappears within a few ns (Fig. 2(c)). At present, we cannot say whether there is enough oxygen in the suspension to explain this effect. We believe that determining the fate of the MV-captured electron is a separate research project, ie. beyond the scope of this work.

4. The authors mention that PL intensity does not noticeably change in the studied T window. It is somehow in contradiction with previous reports showing a strong increase in the PL intensity of AIS-based QDs with lowering T (at least between RT and 100-150 K). At the same time, no

data can be found in the paper and SI on the evolution of the intensity and spectral parameters of PL with temperature to make independent conclusions.

This is a very good point and we thank the Reviewer for raising it. It was our omission. In the Supporting Information, we add a figure (Fig. S10) presenting the PL intensity dependence on the temperature for both samples and add the relevant discussion. In short, between 5 and 100 K, where the PL lifetime changes by a factor of 5, the PL intensity changes by less than 10%. The Reviewer is right that above 150 K (100 K) for sample A1 (A2), the PL intensity starts to drop indicating increased nonradiative decay rate. However, in this temperature range, the change in lifetime is small and completely accounted for by our equilibrium formula (eq. 5 of the main text).

5. The authors dismiss the DAP model and suggest a mechanism explaining PL as a result of the radiative recombination between delocalized electron and trapped hole. Please comment on the origin of the large spectral width of the PL band in the frame of this model.

We thank the Reviewer for another good suggestion. We add a few sentences in which we provide the interpretation of the PL properties observed by many authors on alloyed QDs. On page 12, we add

Within this picture, the common PL properties of alloyed QDs, shown in Fig. 2(a) and 2(b), can be explained. The large Stokes shifts are a consequence of hole localization at intra-gap states. Large PL linewidths are attributed to a distribution of localization energies and a strong electron-phonon coupling. Finally, the spectrally dependent PL decays reflect a correlation between localization energies and positions: traps located closer to the surface are deeper due to an enhanced electron-phonon coupling. The electron-hole wavefunction overlap for localization closer to surface is smaller than for holes localized at the QD center leading to longer and shorter lifetimes, respectively.

6. No clear reasons are provided for the sample A2? Was it used to compare nanorods with QDs? So, what is the outcome? Are the identical? Then A2 becomes redundant.

We thank the Reviewer for this question. Indeed, other Reviewers have raised similar ones indicating that we didn’t explain the reasons for sample A2 well enough. Since questions of other Reviewers regarding sample A2 are similar, we answer all of them together below.

Additional Questions:

Urgency: Moderate

Significance: High

Novelty: High

Scholarly Presentation: Top 10%

Is the paper likely to interest a substantial number of physical chemists, not just specialists working in the authors' area of research?: Yes

Reviewer: 2

Recommendation: This paper may be publishable, but major revision is needed; I would like to be invited to review any future revision.

Comments:
The authors prepared the two types of Ag-In-Zn-S QDs separately with spherical shape (sample A1) and rod shape (sample A2), and these samples were analyzed by transient absorption spectroscopy. Contrary to the conventional understanding that the broad emission of Ag-In-Zn-S QDs is due to D-A pairs, the results suggest the possibility that delocalized electrons and localized holes are responsible for the majority of the emission. From the results of temperature-dependent PL decay measurements, the relationships between the spatial distribution and energy of the acceptor levels within the particles have been considered.
Overall, the analysis and discussion are detailed, providing some new insights, and the reviewer considers the paper to be acceptable for publication in J. Phys. Chem. Lett.

We thank Reviewer 2 for the positive evaluation of our work.

However, the mention of sample A2 is mostly limited to the supporting information, with references to it “being analogous to sample A1” in the main text, making its significance unclear. Recently, band-edge emission by shell formation of group 13-16 semiconductors has been reported, and it has been shown that the shape and compositional distribution within particles significantly affect the band structure and defect levels. If there are optical differences between samples A1 and A2 other than shape and composition, they should be clearly stated in the main text.

We thank the Reviewer for pointing out our insufficient discussion of the results obtained on sample A2. Since other Reviewers raised similar questions, we answer them together below.

In addition, please revise the manuscript with particular attention to the following points:

The authors consider that the samples are cation-rich by assuming charge neutrality. However, is there a possibility of oxygen (oxides)? How was the composition determined?

We discard the possibility of formation of oxides based on XRD and HR-TEM analyses of the studied samples presented in Ref. 11. The measured diffraction patterns reveal exclusively diffraction peaks at intermediate positions between the characteristic ones for orthorhombic $AgInS_2$ and wurtzite ZnS. This observation allows us to conclude that the samples consist of alloyed nanocrystals with no measurable presence of other phases. This conclusion is confirmed by the analysis of interplanar distances measured from HR-TEM images.

The nanocrystal composition was determined via analysis of energy dispersive X-ray spectroscopy (EDS). Please see the Supporting Information, Fig. S2. We clarify this in the revised first paragraph after introduction, on page 5:

Briefly, as shown by x-ray diffraction studies and high resolution transmission electron microscopy, the QD crystal structure forms an alloy of orthorhombic $AgInS_2$ and wurtzite ZnS. No other phases were detected. The chemical composition for A1 and A2, determined via energy-dispersive x-ray spectroscopy, can be written as $AgIn_{1.5}Zn_{1.9}S_{3.6(4.6)}$ and $AgIn_{1.5}Zn_{4.4}S_{6.8(7.1)}$, respectively.

In eq.1, should the last term (Coulomb interaction) be a negative value? Hence, the reviewer considers that "the decays at, respectively, high and low energies, probe donor-acceptor pairs closer and further apart" is opposite.

We kindly note that the Coulomb interaction occurs between ionized donors and acceptors, i.e., in the final state of the DAP transition. Hence, the Coulomb term has a positive sign. See, e.g., Refs. 17-21 and Yu & Cardona, Fundamentals of Semiconductors, 4th ed. 2010, eq. 7.17 on page 356.

In Figure 2c, the TA spectrum has been color-coded in certain wavelength ranges, but in my printing environment, the spectrum is obscured (totally veiled). It seems that the image is provided as a metafile, but to avoid this issue, it might be better to provide it as an image file. Additionally, the color scheme of the graph is not very easy to see.

We apologize for the inconvenience and this omission. In the revised manuscript, we produced the figures with a proper vector graphics software to avoid these kinds of issues. We also checked the visibility with different pdf viewers. We hope that in the present version, the figure is clearly visible.
We also corrected Fig. S3, which previously was produced in the same way as Fig. 2.

In the regions PA1 and PA2 of Figure 2c, for example, the graph at 0.4 ps does not appear to change by 0.5 mOD. The difference with Figure 2d needs to be explained.

We thank the Reviewer for pointing out our mistake. To enable comparison of the dynamics to XB, PA1 and PA2 transients are multiplied by 10. We forgot to mention this fact in the legend of Fig. 1. (The multiplication is mentioned in the legend of Fig. S3.) To avoid confusion, to the caption of Fig. 1, we add

Note that PA1 and PA2 transients were multiplied by 10 to enable the comparison with the XB transient.

The format of the figure numbers does not match the format of the figure numbers in the text. In the figures, they are written in lowercase letters with parentheses (a), (b), (c), while in the text in some parts they are written as 2A, 2B, 2C in uppercase.

In the revised version of the manuscript, the figures are uniformly labeled in lowercase.

"The presence of the long-lived delocalized carrier population and the lack of a slow rise dynamics in the PL transient rules out the DAP recombination as the PL mechanism." Is the time resolution of PL decay measurement sufficient to distinguish the difference in the lifetime of 36 ns of XB?

The time resolution of the PL decay measurements is about 1 ns, see the Supporting Information, Section S1.7. On the other hand, the decay of the XB, which follows the decay of the delocalized electron population, is much longer than 3.6 ns, i.e., the time window available to TA. The decay of XB is thus in tens of ns or longer. The comparison of the PL transient with the instrument response function (IRF) of the TCSPC detection shown in Fig. 2(e) indicates that the PL rise time is similar to the IRF rise time, which is sub-ns. Overall, these comparisons indicate that the PL rise time is at least two orders of magnitude shorter than the decay of delocalized electron population.

Looking at the temperature dependence of the PL decay in Figure 4b, there appear to be at least two lifetime components, one of which appears to become longer with temperature. Since the

fitting was performed using eq. 3, which relates to the distance between the quantum dot center and the acceptor site, it appears that the single tau 0 value is shortening (accelerated emission decay, Figure 4c) with temperature. However, is there a component that shows the opposite behavior?

We thank the Reviewer for this question. In the previous version of the manuscript we ignored this issue for the sake of simplicity, but as the question indicates, it may have caused confusion. It is true that looking at the data in a long time range, there seems to be a short decay component that slows down with increasing temperature. We believe that it arises because the initial part of the decay is not well visible. Below, we plot the first microsecond of the decays shown in Fig. 4 plus one decay at still higher temperature. This is raw data normalized to maximum signal. The figure shows that, indeed, there is an initial, fast decay component whose amplitude strongly decreases with increasing temperature. We attribute this fast decay to thermalization effects (see, e.g., Labeau *et al.* [Phys. Rev. Lett. 2003, 90, 257404], Biadala et al. [ACS Nano 2016, 10, 3356]). To extract the lifetime of thermalized electron-hole pairs, we start the fitting at a delay of 80 ns (indicated by the arrow in the figure). In that range, clearly, the PL decay monotonically accelerates with increasing the temperature as recovered by the temperature dependence of τ_0 lifetimes plotted in Fig. 4.

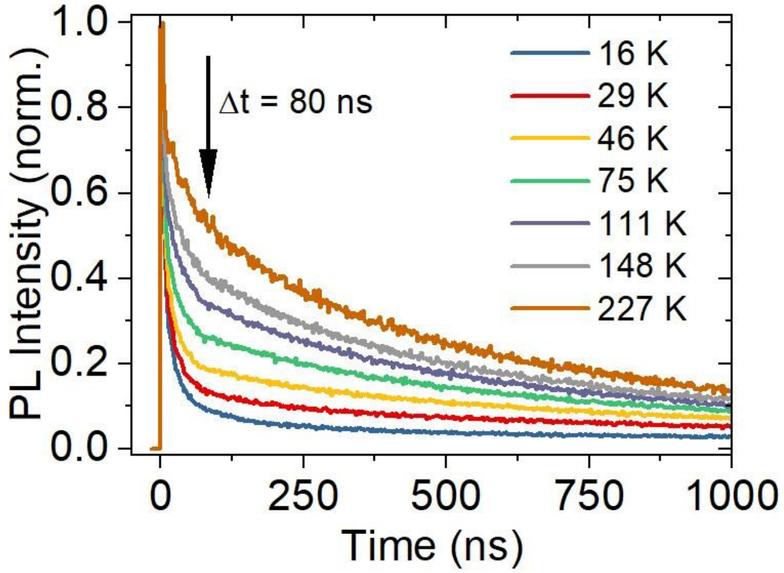

To clarify, we add in the main text, page 14:

In this temperature range, we observe an initial, short decay component visible up to ~40 ns and a long decay component, which accelerates as the temperature increases. The amplitude of the fast component decreases with temperature indicating thermalization effects. We perform fitting of long decays with eq. 5 starting at a delay of 80 ns…

Additional Questions:
Urgency: Moderate

Significance: High

Novelty: Moderate

Scholarly Presentation: High

Is the paper likely to interest a substantial number of physical chemists, not just specialists working in the authors' area of research?: Yes

Reviewer: 3

Recommendation: This paper is publishable subject to minor revisions noted. Further review is not needed.

Comments:
This manuscript presents transient absorption and temperature-dependent PL dynamics data of Ag-In-Zn-S nanocrystals that are consistent with a decay mechanism that involves a delocalized quantum-confined electron recombining with a localized hole. This mechanism contrasts with the alternative donor-acceptor-pair (DAP) mechanism in which both the electron and hole are localized to defect states. The data and analysis are clearly presented with a cohesive argument that clarifies the mechanism of PL decay in quaternary sulfide systems. This manuscript therefore represents an informative addition to the literature on this class of materials. I recommend publication after minor revisions to address the following two points.

We appreciate the Reviewer's positive evaluation of our work.

1. The introduction of the paper contrasts the DAP mechanism with the free-to-bound mechanism and states that the "prevailing view in the literature is that PL occurring [in Ag-In-Zn-S QDs] as a result of the DAP recombination..". However, this statement has no specific references

associated with it. The introduction could be strengthened by including clearer references to specific reports that favor the DAP mechanism for Ag-In-Zn-S nanocrystals.

We have added the relevant references where this issue is first mentioned – at the beginning of the paragraph the Reviewer is quoting:

…circumstantial evidence, it is widely assumed that in quaternary QDs the DAP mechanism is responsible for the PL.11,17-19,22-24

2. The paper presents spectral data for two different samples: spherical particles (A1) and rod-shaped particles (A2). All of the data in the main text are for sample A1 and the data for A2 are only included in the supporting information. The only mention of the A2 sample in the main text is that it has analogous room temperature transient absorption spectral properties to A1. The manuscript should elaborate on the significance of the data from sample A2, particularly the temperature dependent PL dynamics. Is the value of the dark-bright splitting obtained from the A2 sample similar to that for the A1 sample? How was the model described by equations 3 and 4 adapted for a rod-shaped particle given that it assumes a spherical distribution of hole localization states?

We thank the Reviewer for this question. All three Reviewers indicated that we haven't explained the rationale for sample A2 and the obtained results. Therefore, after discussing the theoretical predictions concerning DAP on page 16, we have added a thorough discussion of sample A2. We copy it below. Also, we modified Fig. 4 to include the temperature dependent PL dynamics as requested by Reviewer
3.

Let us now discuss the results obtained on the sample A2 containing rod-shaped QDs. In this case, the mean radius of ~ 2 nm is smaller, while the mean length of 9 nm is larger than a_C estimated above. Despite the different morphologies, we find that the room temperature TA and PL dynamics are quantitatively similar for samples A1 and A2 (compare Figs. 2 and S3). Crucially, in A2 as in A1, XB decay is not accompanied with the rise in PL (see Fig. S3(e)) indicating that delocalized electrons participate in the PL process. Moreover, room temperature PL transients and temperature dependence of PL transients measured for A2 and plotted in Figs. 4(e) and 4(f), respectively, are quantitatively similar to the respective transients for A1 (Figs. 4(b) and 4(c)). These comparisons indicate that in sample A2, as in A1, the PL results from a recombination of a delocalized electron with a localized hole.

To evaluate τ_0, we derive analogs of eqs. 3 and 4 taking into account the rod shape (see the Supporting Information, Section S10). We find that the model curve fits the measured PL transients well, see Fig. S11. The temperature dependence of τ0 together with a fit using eq. 5 is shown in Fig. S12. The obtained bright-dark splitting is 11.0 meV, i.e., almost twice larger than for the sample A1. However, the volume of QDs in A2 is on average 16 times larger than QD volume in A1. We therefore expected a smaller splitting for A2 than for A1 because of a strongly reduced spatial overlap of electron and hole wave functions. To explain this discrepancy, we recall that weaker quantization along the nanorod long axis promotes strong electronic correlations. As a consequence, the ground electron-hole pair state contains admixtures of higher electron states. Since the hole is localized, the correlations lead to squeezing of the pair wavefunction along the long axis compared to the electron wavefunction. In other words, the effective volume occupied by the pair is smaller than the nanorod volume. A similar, albeit weaker, effect of electronic correlations in $CuInS_2$ QDs was explored in Ref. 29. Since detailed accounting for correlations is beyond the scope of the present work, we estimate τ0 using a simplified approach. We assume that the electron wavefunction becomes shrunk to a sphere with a radius equal to the nanorod radius. This assumption may overestimate the effect of correlations but allows us to use eqs. 3 and 4 to extract τ_0 by fitting the PL transients. The result for the room temperature PL transient is shown in Fig. 4(d) and example fits for other temperatures are shown in Fig. S13. The temperature dependence of τ_0 together with a fit using eq. 5 is plotted in Fig. 4(f). The evaluated bright-dark splitting is 8.7 meV. This value is also larger than for sample A1, but now we can attribute it to the correlation-driven shrinkage of the excited state wave function.

Overall, the analysis of PL dynamics presented above supports the conclusion that although the length of the nanorods in sample A2 exceeds aC , the PL is due to a recombination of a delocalized electron with a localized hole.

Additional Questions:
Urgency: High

Significance: Top 10%

Novelty: High

Scholarly Presentation: Top 10%

Is the paper likely to interest a substantial number of physical chemists, not just specialists working in the authors' area of research?: Yes

Once again, we thank all the Reviewers for interesting comments, questions, and suggestions. We believe that after the suggested corrections, our manuscript is improved.

Apart from the above changes, we have also corrected an error in the fitting. In the previous version, we omitted a factor of π in the fitting function. This omission is corrected in the previous version. As a result, all lifetimes are shorter by a factor of $\sim\pi$, which is corrected in the text (page 14). We have exchanged all figures where this fits and fit results were shown: Fig. 4 and Figs. S8 and S9 of the Supporting Information. We note that the new fits are indistinguishable from the previous ones and the omission has in no way impacted the conclusions of this work.

The changes made are indicated in the marked up file attached to the submission.

Sincerely

Łukasz Kłopotowski
(On behalf of all the authors)
